# Comparing active teaching to hybrid lecture-based method for learning radiology basics: A single center controlled study

**DOI:** 10.1016/j.redii.2025.100054

**Published:** 2025-03-08

**Authors:** Fabien de Oliveira, Jean-Paul Beregi, Hugo Potier, Thorgal Brun, Chris Serrand, Julien Frandon

**Affiliations:** a Department of Medical Imaging, Plateforme de recherche en imagerie médicale (PRIM), centre hospitalier universitaire de Nîmes, université de Montpellier, Medical Imaging Group Nîmes, Imagine, 30029, Nîmes, France; b Department of Biostatistics, Clinical Epidemiology, Public Health, and Innovation in Methodology (BESPIM), centre hospitalier universitaire de Nîmes, 30029, Nîmes, France

**Keywords:** Radiology, Lecture-based teaching, Active learning, Controlled trial, Medical education

## Abstract

**Objective:**

There is a lack of knowledge about radiology among medical students at the start of their curriculum. The optimal teaching method for radiological basics remains uncertain. We conducted a controlled trial to compare the effectiveness of full active learning and hybrid lecture-based teaching methods.

**Methods:**

All second-year medical students at Nîmes University Hospital (Nîmes, France) were invited to participate in a training session in the radiology unit. Volunteers were divided into hybrid lecture-based and full active learning groups. The hybrid lecture-based group received a lecture-based session followed by a unit visit, while the full active learning group utilized a structured form with progressive objectives during the visit. Pretests, immediate post-tests, and two-week follow-up tests were conducted. Short-term progression was the primary outcome, with secondary objectives including mid-term acquisition and associated factors.

**Results:**

51 students participated, with 20 in the hybrid lecture-based group and 31 in the full active learning group. Both groups exhibited significant progression between the first and second tests (+8.48 and +2.52 respectively, *p* < 0.01). The hybrid lecture-based group showed significantly greater mean progression (*p* < 0.01). Mid-term results indicated score decrease particularly in the hybrid lecture-based group, but it still maintained significantly superior performance (15.02/20 versus 12.33/20 for full active learning group, *p* < 0.01).

**Conclusion:**

The hybrid pedagogical method yielded superior results in teaching second-year medical students the basics of radiology compared to the full active learning teaching method.

## Highlights

• Dedicated training improves basic radiology knowledge in early medical students.
• Hybrid pedagogy model tends to yield better short- and medium-term outcomes.
• Active learning may not be the most effective approach in all situations.

## 1 Introduction

Despite the crucial role of radiology in patient care and the necessity for physicians to request radiology exams, it remains a relatively unfamiliar medical specialty among medical students at the beginning of their academic curriculum [1,2]. Previous studies have demonstrated the value of introducing students to this specialty during the early years of medical education [3]. As highlighted by recent discussions on interventional radiology education, including its applications in extreme settings, early exposure to the clinical innovations of this field could help overcome knowledge gaps and increase interest in radiology as a career [4,5]. To impart fundamental knowledge and skills in radiology to students, the University Hospital of Nîmes established a half-day training course specifically designed for second-year medical students.

However, determining the most suitable pedagogical method for this purpose is not straightforward. The lecture-based teaching method is the most well-known technique [6]. It follows a teacher-centered approach, where the instructor delivers information to students who passively listen and take notes [7]. Nevertheless, this teaching method relies on the students' ability to concentrate and has been demonstrated to be less effective for skill acquisition compared to interactive approaches [8,9]. Over the past few decades, several innovative teaching methods have emerged, such as flipped classrooms and active learning approaches [10]. In particular, active learning is a constructivist-based method that involves incorporating activities within traditional lectures, encouraging students to reflect on their actions and engage with the material [11,12]. This student-centered model emphasizes active participation and requires students to be actively involved in the learning process. Its main advantages include improved attention and motivation among students [10]. Active learning has been extensively tested in various fields, including pharmacology, maxillofacial surgery, and science, technology, engineering, and mathematics (STEM), demonstrating significant improvements in learning outcomes [12], [13], [14].

Active learning can also incorporate elements of asynchronous learning, which involves a pedagogical method where interactions between teachers and learners can be time-shifted. This may involve incorporating briefing and debriefing periods as part of the learning process [15].

Although studies have demonstrated the benefits of these methods in other fields, there is limited data available on the outcomes of these techniques in the field of radiology. While there are some publications focusing on radiologists' education [16], there is limited data on the learning of radiology basics among novice students. Additionally, there is a lack of data regarding blended learning models that combine various techniques and their effectiveness in this context.

We conducted a controlled trial to compare the effectiveness of a full active learning (FAL) model with a hybrid learning model (HL) that combines a lecture-based technique with a visit to the radiology unit.

## 2 Material and methods

### 2.1 Study design

This study was a controlled, single center, non-randomized trial conducted at the Univeristy Hospital of Nîmes in April 2023. The data were collected prospectively, and the evaluation was conducted in a blinded manner using computerized processes. All second-year medical students from Nîmes Medical University were invited to participate in a half-day (3 h: 9 am–12 pm or 2 pm–5 pm) training session held in the medical imaging unit.

Students who volunteered to participate were included in the study. To ensure manageable group sizes, different groups were planned, with no more than 20 students in each group. After registration, students were assigned to the groups by alternating their placement alphabetically (e.g., the first student to the HL group, the second to the FAL group, and so on), ensuring equal numbers in both groups.

A 15-minute briefing and debriefing session was conducted by a single radiologist from the unit (FO) for all groups, allowing students to ask questions. The briefing covered the structure of the training (including an explanation of the form and available resources for the FAL group), an introduction to the objectives of the session, and a brief overview of radiological modalities (CT, MR). Before the start of the pedagogic session, all students in each group completed a pre-test upon their arrival at the unit. This test was created using the Redcap platform and students completed it online via a personalized link (supplementary appendix 1). The completion time for this test was limited to 10 min.

The same test was administered immediately after the session, before students left the unit, and again 2 weeks after the training session at their homes, online. The same amount of time (10 min) was allotted for these tests as for the pre-test. For the 2-week post-tests, students were instructed to answer the test individually, and the completion time was limited to 10 min. An internet link was made available from 6 pm to 6 am to minimize potential biases. Students were not informed that the tests would be the same before and after the training.

### 2.2 Hybrid learning group

After completing the pre-test, the students in the HL group participated in a one-hour lecture course delivered by the same radiologist from the unit (FO) who conducted the briefing, ensuring consistency and minimizing information bias. Following the lecture, the students were divided into subgroups of a maximum of three students each and were assigned to different locations within the unit where medical imaging devices such as CT or MR were available.

The students were instructed to closely follow the technicians and medical staff present at each site and actively engage by asking questions about the process and rationale behind the various examinations. They were asked to switch between MR and CT sites after half an hour. During this practical session lasting one hour, the students were given autonomy to explore and learn.

### 2.3 Full active learning group

After completing the pre-test, the students in the group were provided with an imaging exam appropriation form (supplementary appendix 2) to complete. During the briefing time, they were given explanations and instructions on how to complete the form. The students were instructed to closely observe and follow the entire patient journey, starting from their arrival in the department, through the preparation process with the technician, the image acquisition, and finally, the interpretation provided by a radiologist from the unit. The form also included online references for accessing additional information. The form aimed to teach the learning objectives, focusing on the importance of including patient and requester information, evaluating the relevance of exams, understanding contraindications, contrast agents, and radiation protection, as well as discussing patient experience. Students were asked to complete the form by consulting radiographers, radiologists, or online resources for any missing information. The resources included an online guide for requesting imaging studies from the French society of radiology and a course material outlining the learning objectives.

Similar to the HL group, the students were divided into subgroups of a maximum of three students each. They were distributed within the department, following the same distribution pattern as the HL group. The students were asked to switch between MR and CT after one hour, extending the practical part of the session to two hours.

### 2.4 Test and pedagogical objectives

Upon their arrival, the students were first asked demographic questions, including age, sex, and academic background. Following this, they completed a test assessing their knowledge of radiology. The questions for the test were selected based on the pedagogical objectives established for second-year medical students. These objectives can be categorized under the first two levels of Miller's pyramid [17]:

1. Knows: Understanding the contraindications of CT and MR exams, as well as the adverse effects associated with the contrast agents used in CT and MR.
2. Knows how: Explaining to patients how an MRI and CT exam are conducted and understanding the context of responsibility in medical imaging.

The test consisted of multiple choice questions (MCQ), simple choice questions (SCQ), and short answer questions (SAQ). Students were given 10 min to complete the test. The correction of the test was based on a predefined scoring rubric (supplementary appendix 3). The analysis of the test results was conducted blindly, without knowledge of the groups, individual students, or previous test results. The result was reported on a scale of 20, which corresponds to what is usually done in the French educational system.

### 2.5 Endpoints and assessments

The primary endpoint of the study was the mean difference in test progression between the two groups from the first test. The secondary endpoints included the difference in test progression between the groups at the 2-week mark, as well as exploring any potential links between the results and the students' academic background, age, and sex.

The students' academic background was assessed by two possibilities: Parcours Accès Santé Spécifique (PASS), which indicates that the students had only one year of university education between their high school diploma and the second year of medical school, and "cursus other than PASS," which means that the student had at least two years of university education before entering the second year of medical school.

In addition to the test results, the study also assessed the satisfaction of the students and their perceived utility of the training session. These aspects were measured using a 0 to 10 scale, with the students providing their ratings at the end of the post-test.

### 2.6 Statistical analysis

Quantitative variables were presented with their mean and standard deviation or median with interquartile range, depending on their distribution. To compare these variables, either the Student *t*-test or the Mann-Whitney test was used, depending on the distribution of the data.

Qualitative variables were presented with their frequency and associated proportions. The comparison of these variables was performed using the Chi-squared test, or the Fisher's exact test if the validity conditions for the Chi-squared test were not met.

The normality of continuous variables was assessed graphically.

Factors associated with test results were investigated using multivariable linear regression models. The grades at each test, as well as the changes in grades between the pretest, immediate post-test, and later assessment, were explored. The variables included in each model were the sex and age of the student, group assignment (FAL or HL), other obtained diplomas, the student academic background (access to 2nd year via PASS or not), presence of a radiologist in the family, previous radiology internship, and interest in becoming a radiologist. The estimated changes in grades were presented with their corresponding 95 % confidence intervals.

All analyses were performed at a conventional two-sided alpha level of 0.05 using SAS 9.4 software.

## 3 Results

### 3.1 Characteristics of students

**Fig. 1:**
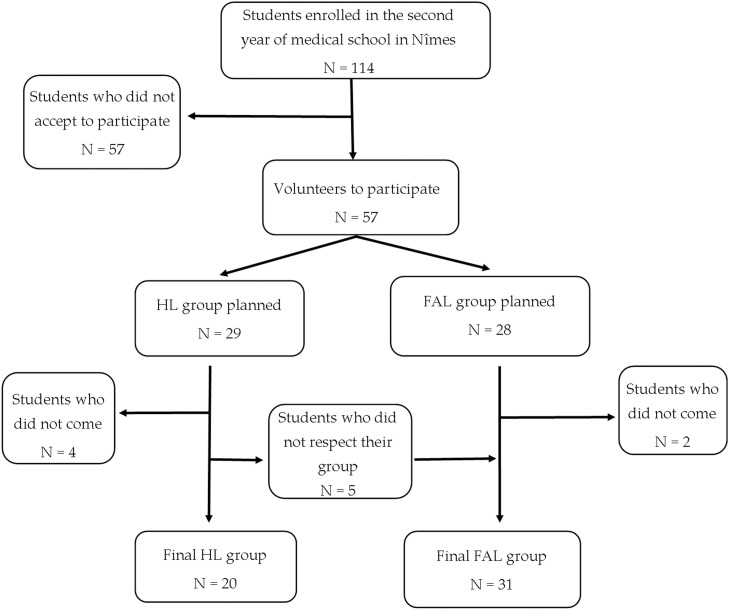
Single center study to compare the effectiveness of full active learning (FAL) and hybrid lecture-based (HL) teaching methods: population selection.

The participation rate was 44.7 %. Based on the registration data, initially, 57 students volunteered to participate and were divided into three groups of 14 students each, with one group consisting of 15 students. However, due to some students not attending the session or not adhering to their assigned groups, the composition of the groups was modified. The hybrid learning (HL) group ended up with a first subgroup of nine students and a second subgroup of 11 students, while the full active learning (FAL) group had a first subgroup of 18 students and a second subgroup of 13 students. Consequently, the final participation consisted of 51 students (Fig. 1).

Among these students, there were 76 % women overall, 85 % in the HL group and 71 % in the FAL group. Mean age was 20.0 years overall, 19.8 years in the HL group and 20.1 years in the FAL group.

In the HL group, no student had an academic degree prior to enrolling in medical school. In the FAL group, it concerned 13 % of students (Table 1).

**Table 1:** Single center study to compare the effectiveness of full active learning (FAL) and hybrid lecture-based (HL) teaching methods: Students’ characteristics.

		FAL *N* (%)	HL *N* (%)	*p*
Sex	Women	22 (70.97)	17 (85.00)	0.32
	Men	9 (29.03)	3 (15.00)	
Age	Mean (± SD)	20.13 (±1.48)	19.75 (±1.02)	0.30
	Median (IQR)	20.00 (19.00; 20.00)	19.50 (19.00; 20.00)	
Access to 2nd year via PASS	Yes	14 (45.16)	6 (30.00)	0.28
	No	17 (54.84)	14 (70.00)	
Prior academic degree	Yes	4 (12.90)	0 (0.00)	0.15
	No	27 (87.10)	20 (100.00)	
Aware of the radiology specialty	Yes	8 (25.81)	8 (40.00)	0.29
	No	23 (74.19)	12 (60.00)	
Already visited a radiology unit	Yes	11 (35.48)	8 (40.00)	0.74
	No	20 (64.52)	12 (60.00)	

### 3.2 Tests results

Tests results are reported in Table 2.

**Table 2:** Single center study to compare the effectiveness of full active learning and hybrid lecture-based teaching methods: Learning progression and mid-term knowledge maintenance.

		**Total**	**FAL**	**HL**	*p*
*Pre- and post-test results*		*N* = 51	*N* = 31	*N* = 20	
Pretest results	Mean (± SD)	9.20 (±2.88)	9.89 (±2.51)	8.14 (±3.14)	0.06
Post test results	Mean (± SD)	14.06 (±3.05)	12.41 (±2.35)	16.62 (±2.12)	< 0.01
Difference bewteen pre and post test	Mean (± SD)	4.85 (±4.08)	2.52 (±2.66)	8.48 (±3.17)	< 0.01
*Midterm test results*		*N* = 37	*N*= 26	*N* = 11	
Midterm test results	Mean (± SD)	13.13 (±2.31)	12.33 (±2.07)	15.02 (±1.73)	< 0.01
Difference between pretest and midterm test	Mean (± SD)	3.96 (±3.40)	2.67 (±2.78)	7.03 (±2.75)	< 0.01
Difference bewteen posttest and midterm test	Mean (± SD)	−0.63 (±1.97)	0.03 (±1.57)	−2.17 (±2.01)	< 0.01

In the HL group, there was a mean progression of + 8.48 points between first and second test. In the FAL group, mean progression was + 2.52 points. The difference in mean progression between the two groups is statistically significant (*p* < 0.01).

Overall, 37 students completed the midterm test, representing 72.5 % of the total cohort, with 83.9 % in the FAL group and 55.0 % in the HL group. Mean progression between the first test and midterm test was +2.67 points in the FAL group and + 7.03 points in the HL group. The difference in mean progression between the two groups is statistically significant (*p* < 0.01) (Table 2).

### 3.3 Satisfaction and utility perceived

Mean satisfaction score was 9.4/10 in HL group and 8.0/10 in FAL group (*p* < 0.01). Mean utility score was 9.7/10 in HL group and 7.3/10 in FAL group (*p* < 0.01).

### 3.4 Link between results and students’ characteristics

Mean pretest score for students coming from PASS cursus was initially 9.6/20 versus 8.6/20 for students not coming from PASS. Male sex was found to be associated with a better initial result (Table 3).

**Table 3:** Single center study to compare the effectiveness of full active learning and hybrid lecture-based teaching methods: Factors associated with pretest results.

	Score difference	Confidence interval	*p*
Age	−0.04	[−0.91; 0.83]	0.9236
Sex (male vs. female)	1.89	[0.04; 3.74]	0.0450
PASS vs no PASS	1.07	[−0.79; 2.94]	0.2516
Prior academic degree	−0.43	[−4.61; 3.74]	0.8355
Interested by radiology	1.23	[−0.62; 3.09]	0.1877
Already visited a radiology unit	0.6	[−1.24; 2.43]	0.5148

Regardless of gender, age and other factors, there was a significant effect of the HL compared to the FAL method, with HL method showing a higher improvement in immediate and medium-term post-test scores compared to the other method. The other variables studied showed no influence on students' progress (Table 4).

**Table 4:** Single center study to compare the effectiveness of full active learning (FAL) and hybrid lecture-based (HL) teaching methods: Factors associated with the evolution between pretest, post-test and mid-term test.

	Evolution between pre- and post tests			Evolution between pretest and midterm test		
	Score difference	Confidence interval	*p*	Score difference	Confidence interval	*p*
Group HL vs. FAL	5.92	[4.1; 7.74]	<0.0001	4.12	[1.73; 6.51]	0.0015
Age	0.04	[−0.9; 0.99]	0.9239	0.16	[−0.92; 1.23]	0.7683
Sex (male vs. female)	−0.54	[−2.55; 1.46]	0.5878	0.06	[−2.37; 2.49]	0.9613
PASS vs. no PASS	−1.26	[−3.29; 0.76]	0.2153	−0.59	[−2.9; 1.72]	0.6043
Prior academic degree	−1.56	[−6.1; 2.98]	0.4924	−2.18	[−7.75; 3.38]	0.4284
Interested by radiology	0.17	[−1.85; 2.18]	0.8687	0.74	[−1.71; 3.2]	0.5411
Already visited a radiology unit	−1.47	[−3.46; 0.52]	0.1443	−0.71	[−3.16; 1.74]	0.5571

## 4 Discussion

We conducted a prospective controlled study to assess the effectiveness of different pedagogical methods in improving the knowledge of radiological basics among medical students at the early stages of their curriculum. Specifically, we compared a student-centered approach primarily based on active learning with a hybrid method that combined a lecture-based course (teacher-centered) and a visit to the radiology unit. Our findings revealed a statistically significant improvement in knowledge for both groups. However, the hybrid group demonstrated significantly better progress compared to the active learning group. We did not observe a significant impact of student academic background on their results, although the small sample size and protocol deviations may have limited our ability to detect such an effect.

The composition of the groups in our study was not directly comparable. Participation was optional, and no true randomization was implemented in the group assignment. This led to different compositions, with the HL group having more PASS program students, while the FAL group had students from various academic backgrounds, including more prior degrees and older students. These composition differences, in terms of academic background and age, may have influenced the study outcomes and should be considered when interpreting the results. Additionally, some students transferred from the HL group to the FAL group; however, this transfer was unlikely to be motivated by a preference for a specific teaching method, as students were unaware of the method assigned to their group. This behavior was likely driven by scheduling convenience or a desire to join friends.

The initial test scores in both the HL and FAL groups were relatively low, with a mean score of 8.1/20 in the HL group and 9.9/20 in the FAL group. These scores indicate that the students had a limited understanding of the basics of radiology. It is important to note that these results were obtained from student volunteers, many of whom were already interested in radiology. Therefore, it is likely that the results for the rest of the class may be even lower.

Despite the low initial scores, all groups demonstrated a significant improvement in the post-test, indicating the effectiveness of our training session in enhancing knowledge. The primary endpoint of our study was to compare the difference in progression between the two groups, and we found that the hybrid group showed a significantly higher level of progression compared to the active learning group.

Although the number of students included in our study was not extensive, we were able to achieve sufficient statistical power to evaluate our primary endpoint and observe statistically significant results favoring the hybrid group. Numerous comparative studies on medical teaching highlight the benefits of student-centered methods. For example, in infectiology, Ghasemzadeh et al. found that student-based teaching yielded better results than teacher-centered instruction, though their study focused on fourth-year medical students and problem-solving methods, unlike our study on radiology basics for second-year medical students [18].

In the field of anatomy, a study with 424 students showed no significant difference between problem-based learning and traditional methods, suggesting that teaching approaches may yield comparable outcomes depending on the subject matter and student population [19]. However, the context of this study differs, and comparisons should be made cautiously. Moreover, a recent meta-analysis on teaching methods in radiology highlighted significant heterogeneity among studies and noted challenges in drawing definitive conclusions [20]. Nevertheless, it suggests that active learning often yields better outcomes compared to passive methods. While our results highlight the benefits of a hybrid approach—combining a lecture with a visit to the radiology unit—this does not entirely contradict the findings of the meta-analysis.

The apparent differences can be attributed to multiple factors. Our younger participants, unfamiliar with active learning methods, may have benefited more from the structured lecture-based approach. Additionally, motivation and maturity likely played a role. As the intervention was optional, unrelated to examinations, and non-credit bearing, students may not have felt compelled to fully engage. In the active learning group, where more effort was required to seek answers, this lack of external motivation may have affected their performance. These results highlight the importance of tailoring teaching methods to the target audience and context, as active learning approaches, while often effective, may not always represent the most suitable choice.

Our findings align with Jordan et al., who found lecture format better than asynchronous, computer-based instruction for teaching acute care to fourth-year medical students. This supports the idea that teaching methods yield different outcomes based on the subject and student population [21]. Notably, there is a lack of studies directly comparing hybrid teaching, like in our study, with active learning. Our research adds valuable insights in this context and underscores the need for further exploration of hybrid teaching effectiveness in medical education.

Several factors explain our results. Firstly, lecture-based teaching in small groups may have improved student attention and engagement, particularly since younger students, with an average age of 20, are more familiar with this method. Given that lectures typically promote superficial, temporary memorization, it is also reasonable to expect that students in the HL group would perform better on the immediate post-test. In contrast, the active learning group was supervised by working professionals, limiting mentoring time. This mirrors real-world conditions, thus adding strength to our study. Additionally, we used a blinded and objective evaluation method, which helped reduce biases and ensured the reliability of the results.

Looking at the mid-term results, we observed a pronounced decrease in the HL group's performance. Despite this decline, their scores remained significantly better than those of the FAL group. One possible reason is regression to the mean; initially, HL group had higher scores, moving closer to the average over time. Conversely, the absence of a significant decline in the FAL group's performance may suggest more stable knowledge retention. As discussed previously, the lecture-based format, which favors immediate memorization, may support this interpretation. However, the superior progression of the HL group, even at the two-week mark, suggests that while the memorization advantage of lectures likely played a role, it cannot fully account for the results difference.

Our study has several limitations to acknowledge. Firstly, there was no true randomization in group assignment, leading to an initial imbalance in test scores that may have influenced results. However, each student acted as their own control, reducing major bias. Additionally, selection bias was present as only volunteering students participated, potentially affecting generalizability. Also, students not being informed about the identical pre-test and post-test introduces recall bias, especially in the active learning group. This raises concerns for the active learning group, as they might have acquired additional knowledge beyond the questionnaire's specific content, unlike the hybrid group, which only received a lecture on the covered topic. We aimed to ensure that students in both groups had access to all the necessary information to answer the test questions, so as not to disadvantage one group over the other. Specifically, the FAL group's appropriation form was designed to address this, and the online resources were intended to provide complementary information. However, we cannot be certain that all students in the FAL group had the opportunity or sufficient time to locate this information, which represents a potential limitation. Moreover, nearly half of the HL group's students were lost at the distant post-test, compared to less than a third for the FAL group, raising questions about student motivation levels, which unfortunately was not assessed in our study. These limitations emphasize the need for further research, addressing these issues and including a broader student population with a more randomized approach.

## 5 Conclusion

Our study shows the effectiveness of a training session in enhancing radiological basics for second-year medical students. The hybrid teaching method appeared to lead to greater knowledge progression compared to the active learning approach. However, these findings should be interpreted with caution, as differences in student motivation, maturity, and familiarity with active learning methods may have influenced the outcomes. Incorporating both didactic instruction and practical exposure may provide a valuable foundation for medical education, but further research with larger and diverse student populations is needed to validate our findings and assess the long-term impact of these teaching approaches.

## Ethics approval and consent to participate

This pedagogical study was conducted in accordance with relevant guidelines and regulations and received approval from an institutional ethics committee (number: 23.08.06). All participants provided informed consent.

## Funding

None.

## Availability of data and materials

The datasets used and analysed during the current study are available from the corresponding author on reasonable request.

## CRediT authorship contribution statement

**Fabien de Oliveira:** Conceptualization, Investigation, Writing – original draft. **Jean-Paul Beregi:** Conceptualization, Methodology, Project administration. **Hugo Potier:** Software, Resources. **Thorgal Brun:** Investigation. **Chris Serrand:** Methodology, Formal analysis. **Julien Frandon:** Conceptualization, Writing – review & editing, Supervision.

## Declaration of competing interest

The authors declare that they have no known competing financial interests or personal relationships that could have appeared to influence the work reported in this paper.

## Appendix Supplementary materials

Image, application 1

Image, application 2

## References

[1] Branstetter B.F., Faix L.E., Humphrey A.L., Schumann J.B. (2007). Preclinical medical student training in radiology: the effect of early exposure. AJR Am J Roentgenol.

[2] Collins J., Dotti S.L., Albanese M.A. (2002). Teaching radiology to medical students: an integrated approach. Acad Radiol.

[3] Vidal V., Jacquier A., Giorgi R., Pineau S., Moulin G., Petit P. (2011). Radiology as seen by medical students]. J Radiol.

[4] Barral M., Razakamanantsoa L., Cornelis F.H. (2021). How to further train medical students in Interventional radiology?. Diagn Interv Imaging.

[5] Frandon J., Soussan J., Vidal V., Nikolov T., Rubino B., Luciani A. (2024). Expanding the horizons of interventional radiology: training analog astronauts for percutaneous drainage in preparation for deep space exploration. Cardiovasc Intervent Radiol.

[6] Sandhu S., O Afifi T., Amara F.M (2012). Theories and practical steps for delivering effective lectures. J Community Med Health Edu.

[7] Barnier G. (2002).

[8] Nilson L.B. (2016).

[9] (2004). Points of view: lectures: can't learn with them, can't learn without them. Cell Biol Educ.

[10] Sivarajah R.T., Curci N.E., Johnson E.M., Lam D.L., Lee J.T., Richardson M.L. (2019). A review of innovative teaching methods. Acad Radiol.

[11] Bonwell C.C., Eison J.A. (1991). Active learning: creating excitement in the classroom. 1991 ASHE-ERIC Higher Education Reports.

[12] Kennedy D.R. (2019). Redesigning a pharmacology course to promote active learning. Am J Pharm Educ.

[13] Milne S., Walshaw E.G., Webster A., Mannion C.J. (2022). Active learning in head and neck trauma: outcomes after an innovative educational course. Br J Oral Maxillofac Surg.

[14] Freeman S., Eddy S.L., McDonough M., Smith M.K., Okoroafor N., Jordt H. (2014). Active learning increases student performance in science, engineering, and mathematics. Proc Natl Acad Sci U S A.

[15] Worthington T. (2013). 2013 8th International Conference on Computer Science & Education.

[16] Klontzas M.E., Karantanas A.H. (2021). Incorporating engineering principles in radiology education: are we ready to face the future?. Diagn Interv Imaging.

[17] Norcini J.J. (2003). Work based assessment. BMJ.

[18] Ghasemzadeh I., Aghamolaei T., Hosseini-Parandar F. (2015). Evaluation of medical students of teacher-based and student-based teaching methods in infectious diseases course. J Med Life.

[19] Prince K.J.A.H., van Mameren H., Hylkema N., Drukker J., Scherpbier A.J.J.A., van der Vleuten C.P.M. (2003). Does problem-based learning lead to deficiencies in basic science knowledge? An empirical case on anatomy. Med Educ.

[20] Wade S.W.T., Velan G.M., Tedla N., Briggs N., Moscova M. (2024). What works in radiology education for medical students: a systematic review and meta-analysis. BMC Med Educ.

[21] Jordan J., Jalali A., Clarke S., Dyne P., Spector T., Coates W. (2013). Asynchronous vs didactic education: it's too early to throw in the towel on tradition. BMC Med Educ.

