# Supplementary material for: Comparing active teaching to hybrid lecture-based method for learning radiology basics: A single center controlled study
Source: Res Diagn Interv Imaging. 2025 Mar 8;13:100054. doi: 10.1016/j.redii.2025.100054 (PMC11930576; doi:10.1016/j.redii.2025.100054)
Supplement: Supplementary file 1 [file mmc1.pptx]

## Slide 1
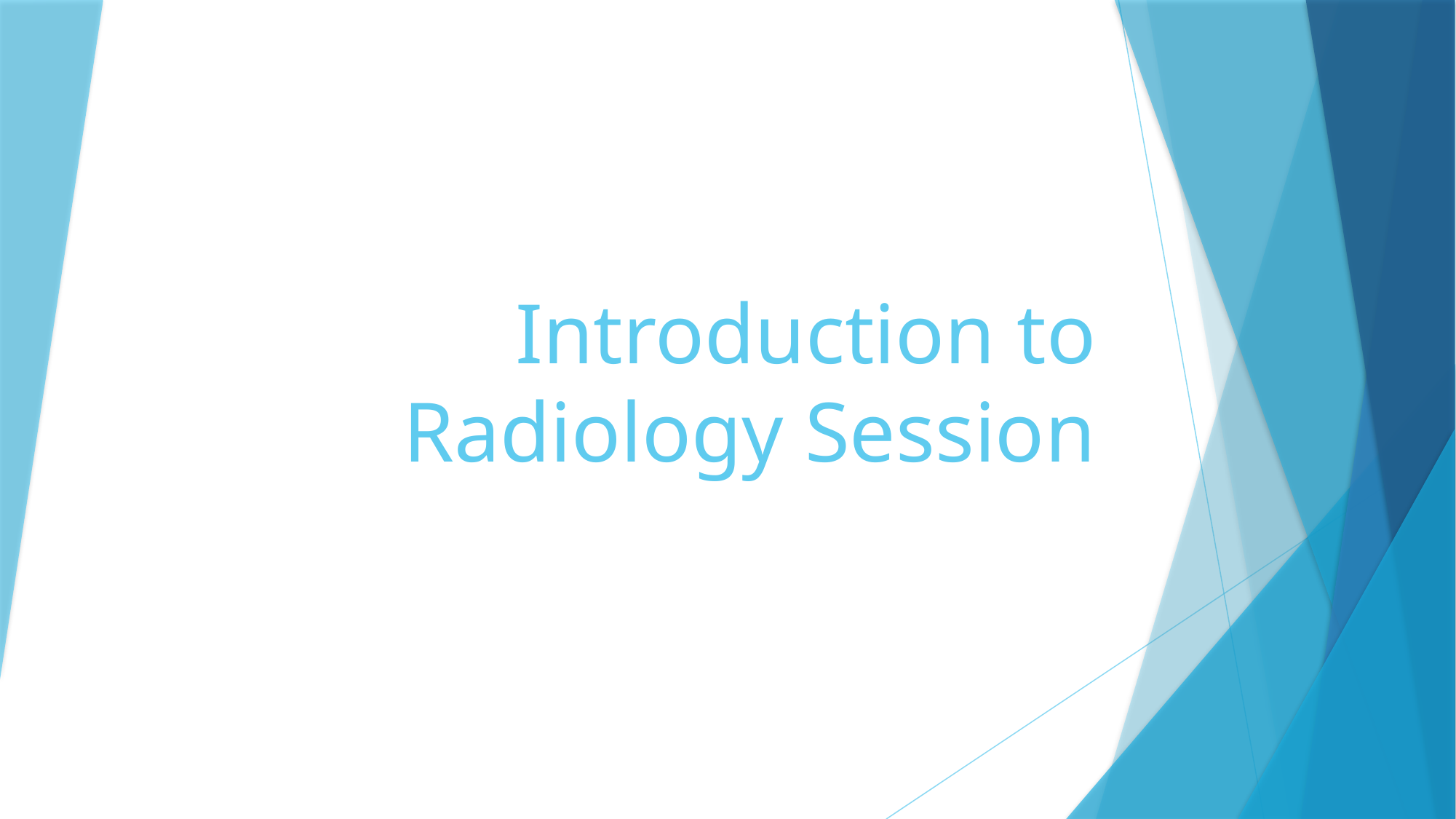

# Introduction to Radiology Session

## Slide 2
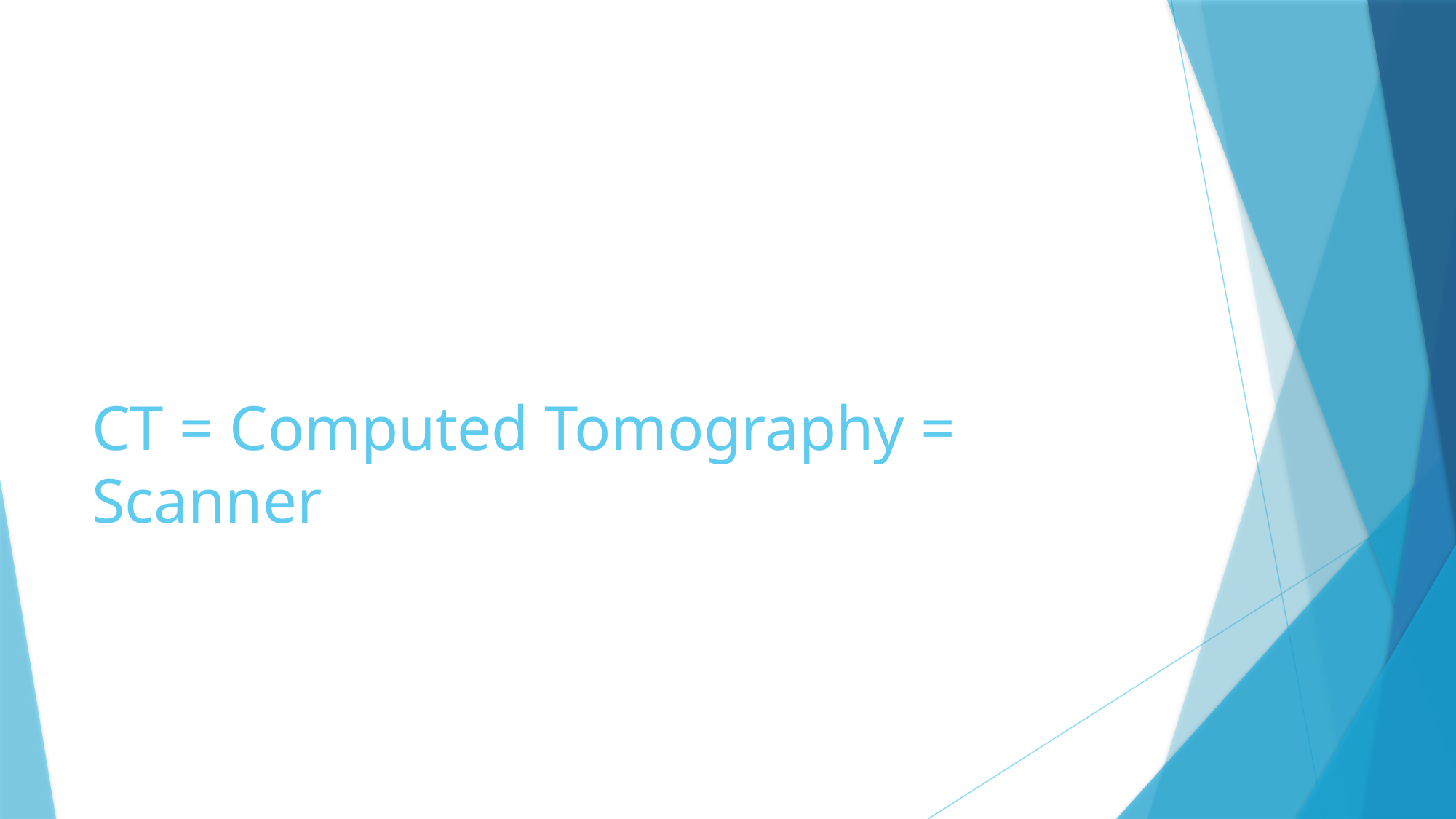

# CT = Computed Tomography = Scanner

## Slide 3
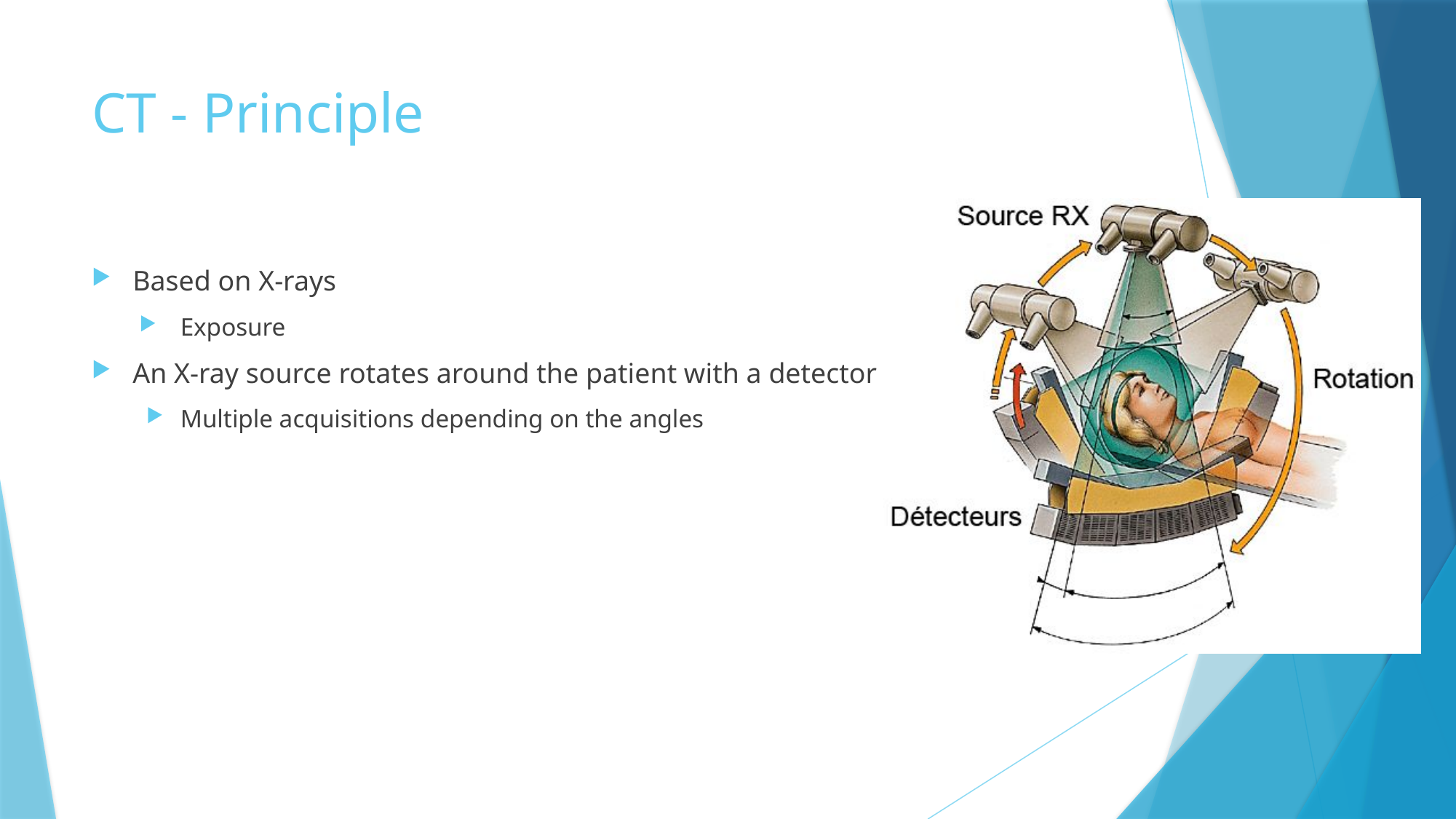

# CT - Principle
Based on X-rays
Exposure
An X-ray source rotates around the patient with a detector
Multiple acquisitions depending on the angles

## Slide 4
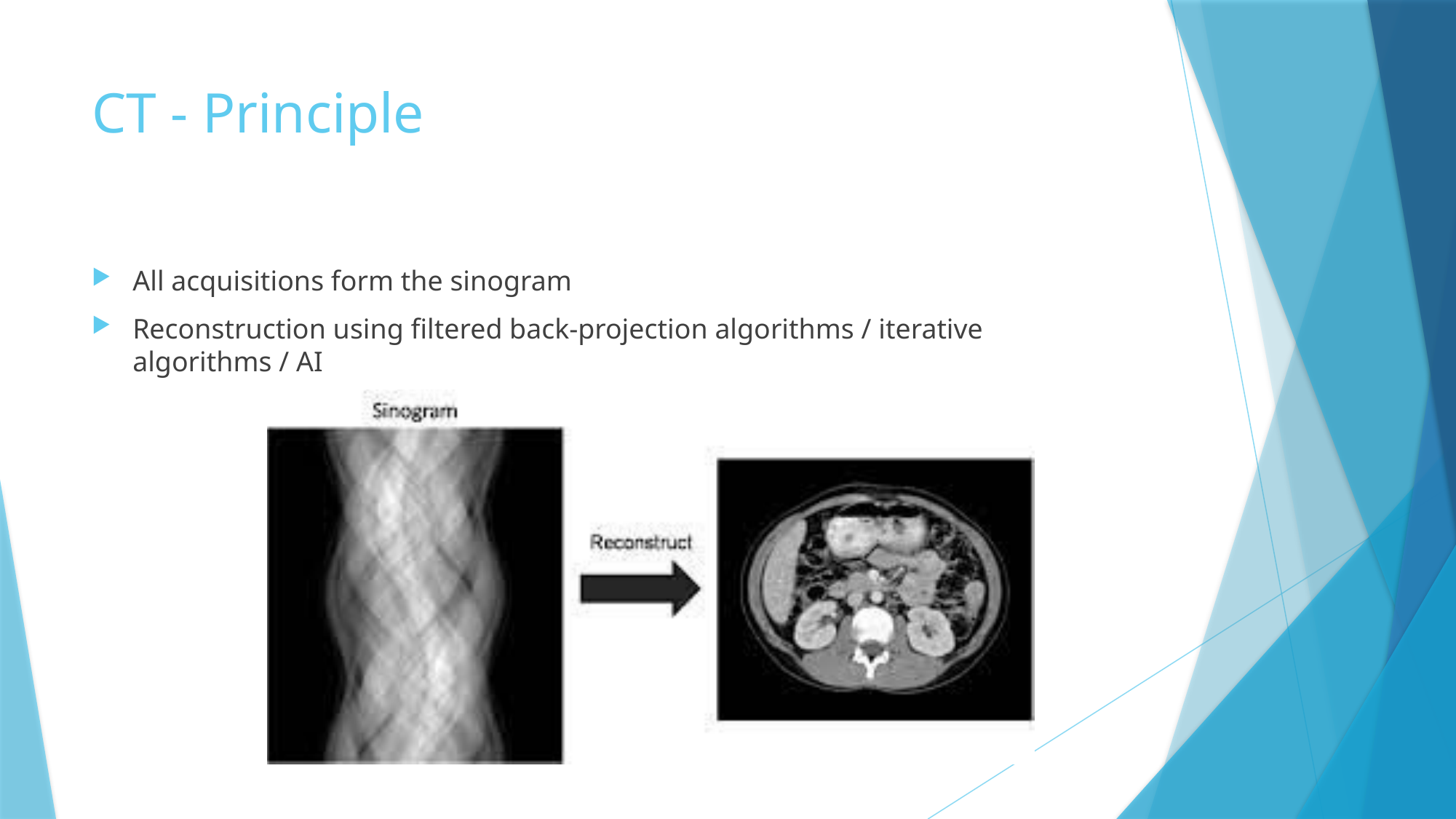

# CT - Principle
All acquisitions form the sinogram
Reconstruction using filtered back-projection algorithms / iterative algorithms / AI

## Slide 5
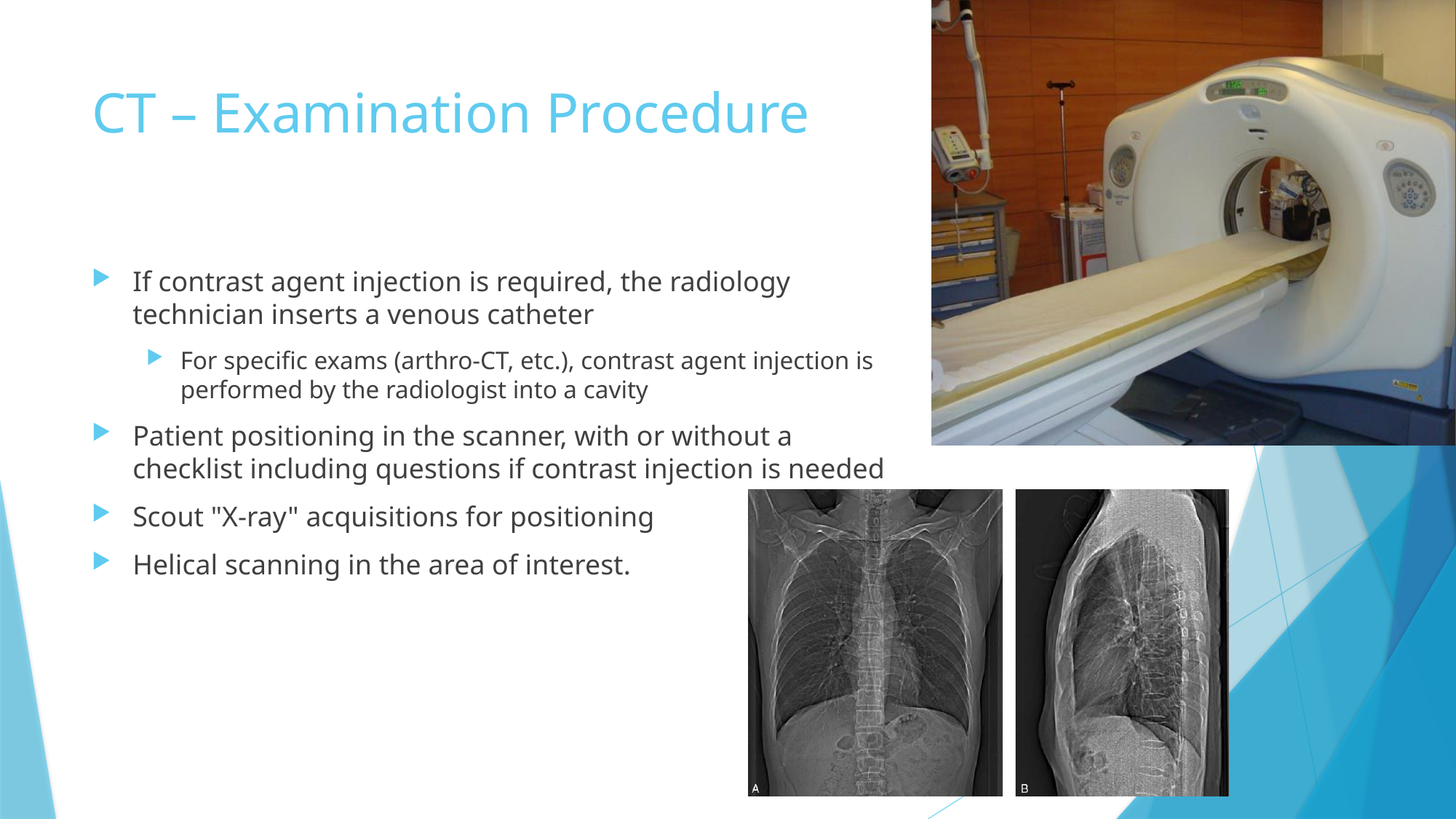

# CT – Examination Procedure
If contrast agent injection is required, the radiology technician inserts a venous catheter
For specific exams (arthro-CT, etc.), contrast agent injection is performed by the radiologist into a cavity
Patient positioning in the scanner, with or without a checklist including questions if contrast injection is needed
Scout "X-ray" acquisitions for positioning
Helical scanning in the area of interest.

## Slide 6
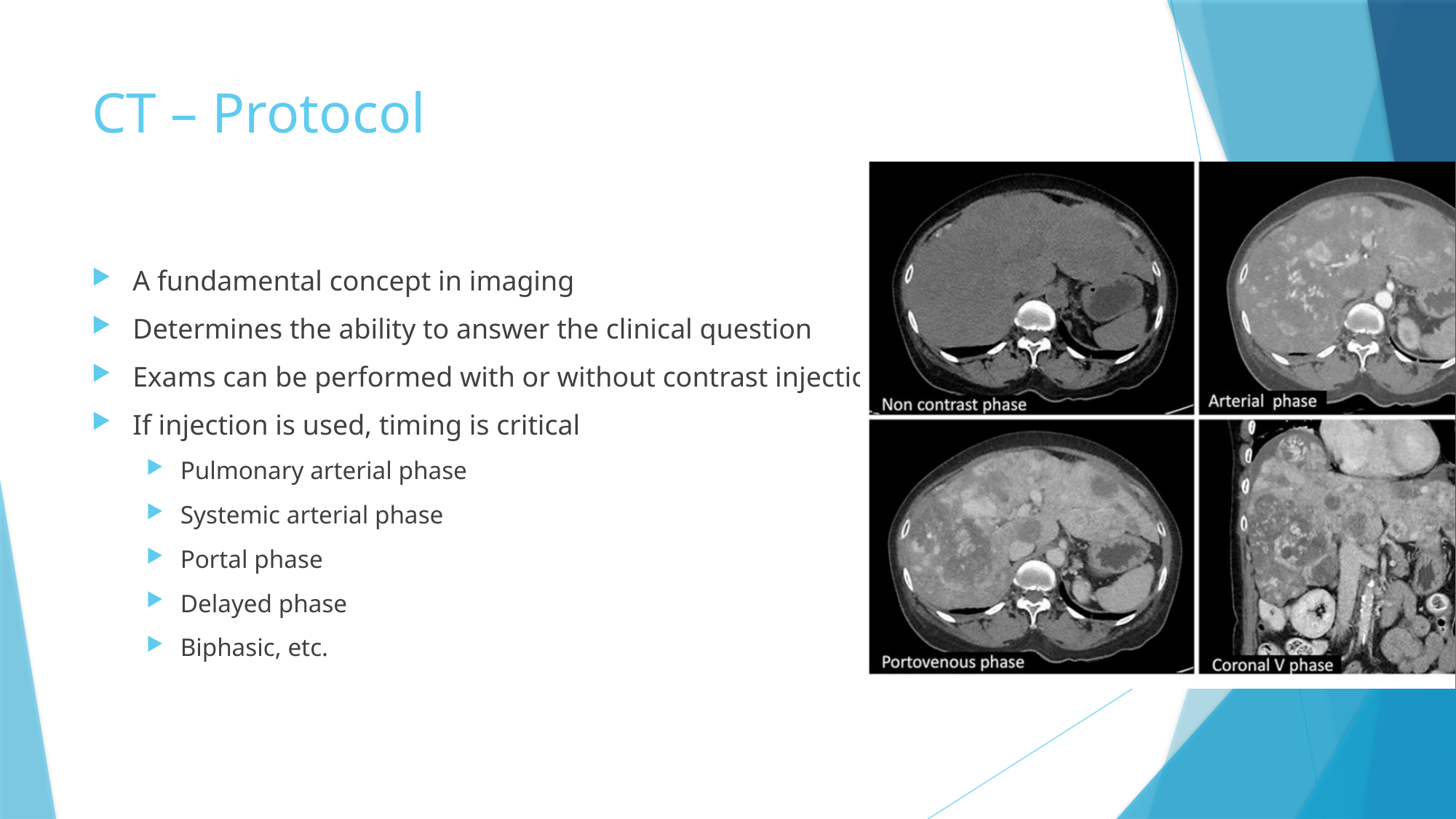

# CT – Protocol
A fundamental concept in imaging
Determines the ability to answer the clinical question
Exams can be performed with or without contrast injection
If injection is used, timing is critical
Pulmonary arterial phase
Systemic arterial phase
Portal phase
Delayed phase
Biphasic, etc.

## Slide 7
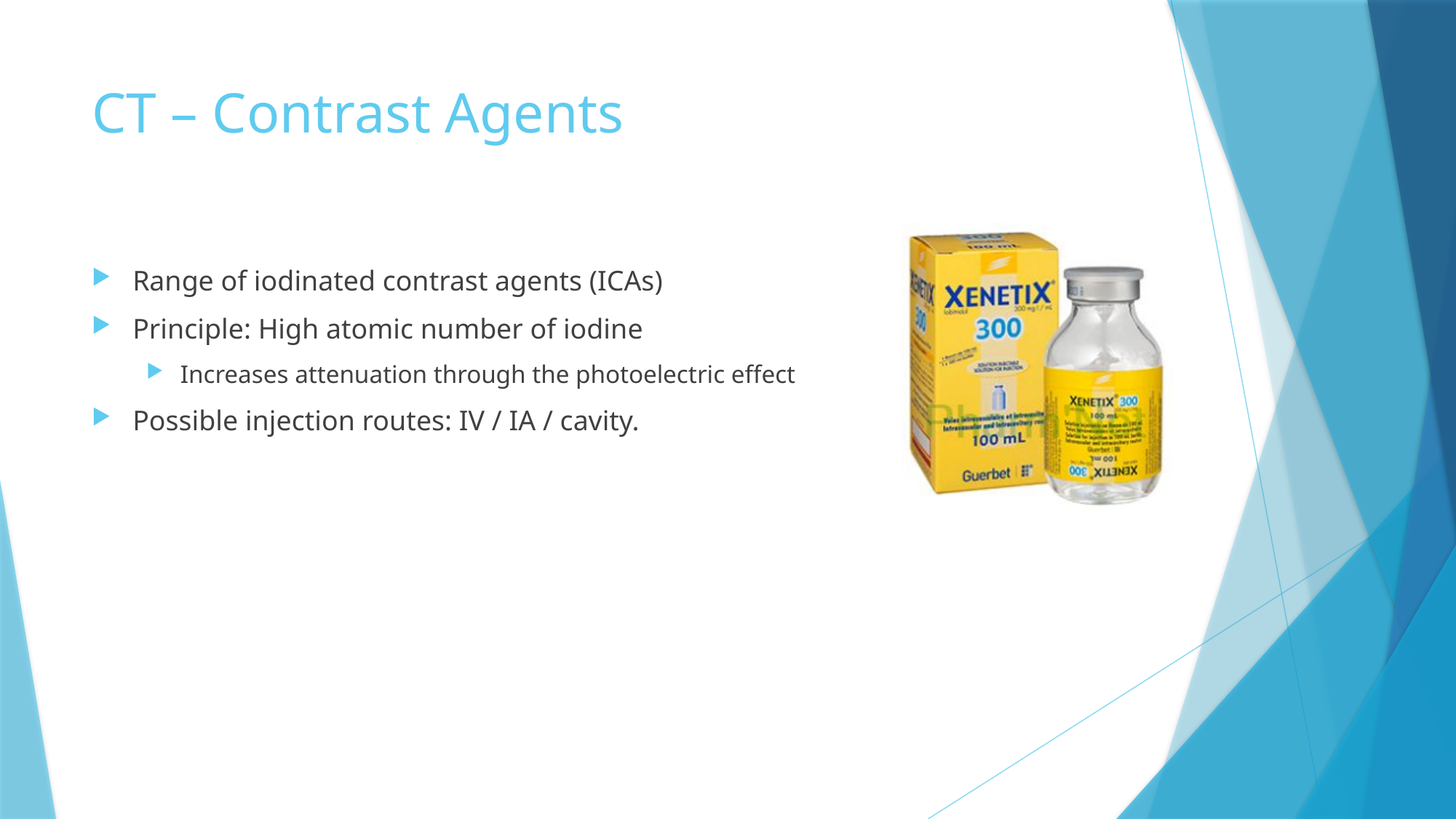

# CT – Contrast Agents
Range of iodinated contrast agents (ICAs)
Principle: High atomic number of iodine
Increases attenuation through the photoelectric effect
Possible injection routes: IV / IA / cavity.

## Slide 8
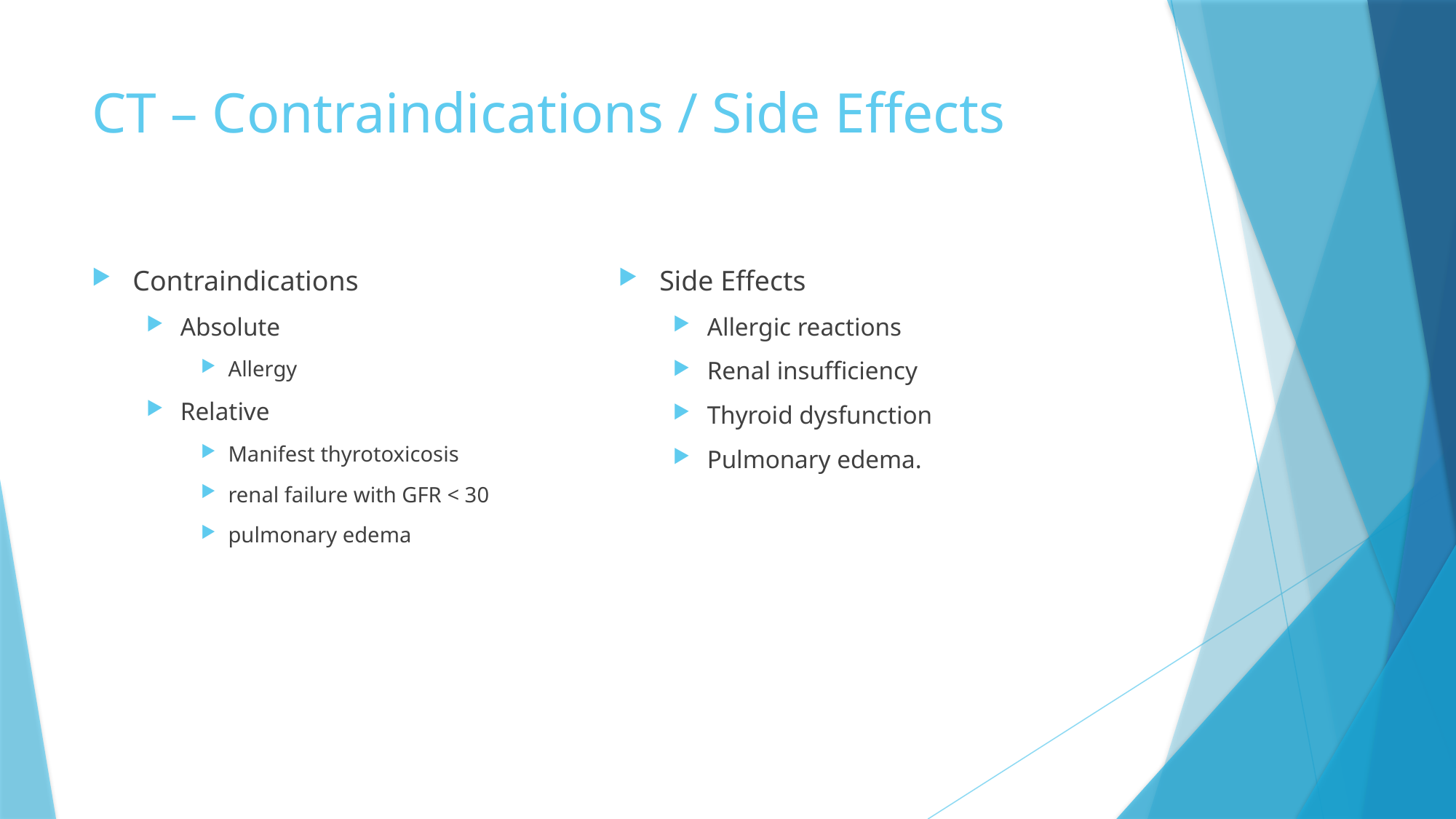

# CT – Contraindications / Side Effects
Contraindications
Absolute
Allergy
Relative
Manifest thyrotoxicosis
renal failure with GFR < 30
pulmonary edema
Side Effects
Allergic reactions
Renal insufficiency
Thyroid dysfunction
Pulmonary edema.

## Slide 9
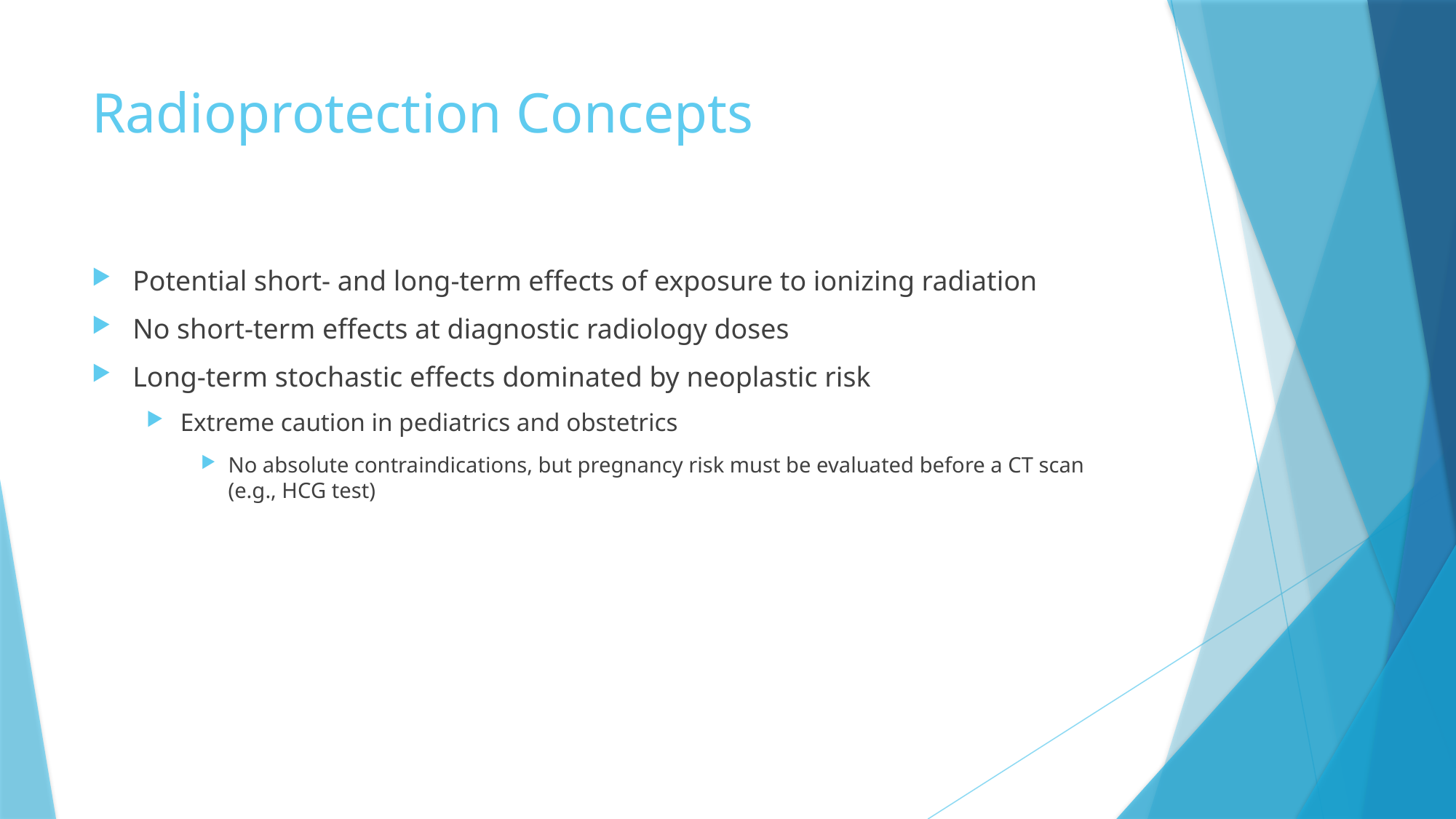

# Radioprotection Concepts
Potential short- and long-term effects of exposure to ionizing radiation
No short-term effects at diagnostic radiology doses
Long-term stochastic effects dominated by neoplastic risk
Extreme caution in pediatrics and obstetrics
No absolute contraindications, but pregnancy risk must be evaluated before a CT scan (e.g., HCG test)

## Slide 10
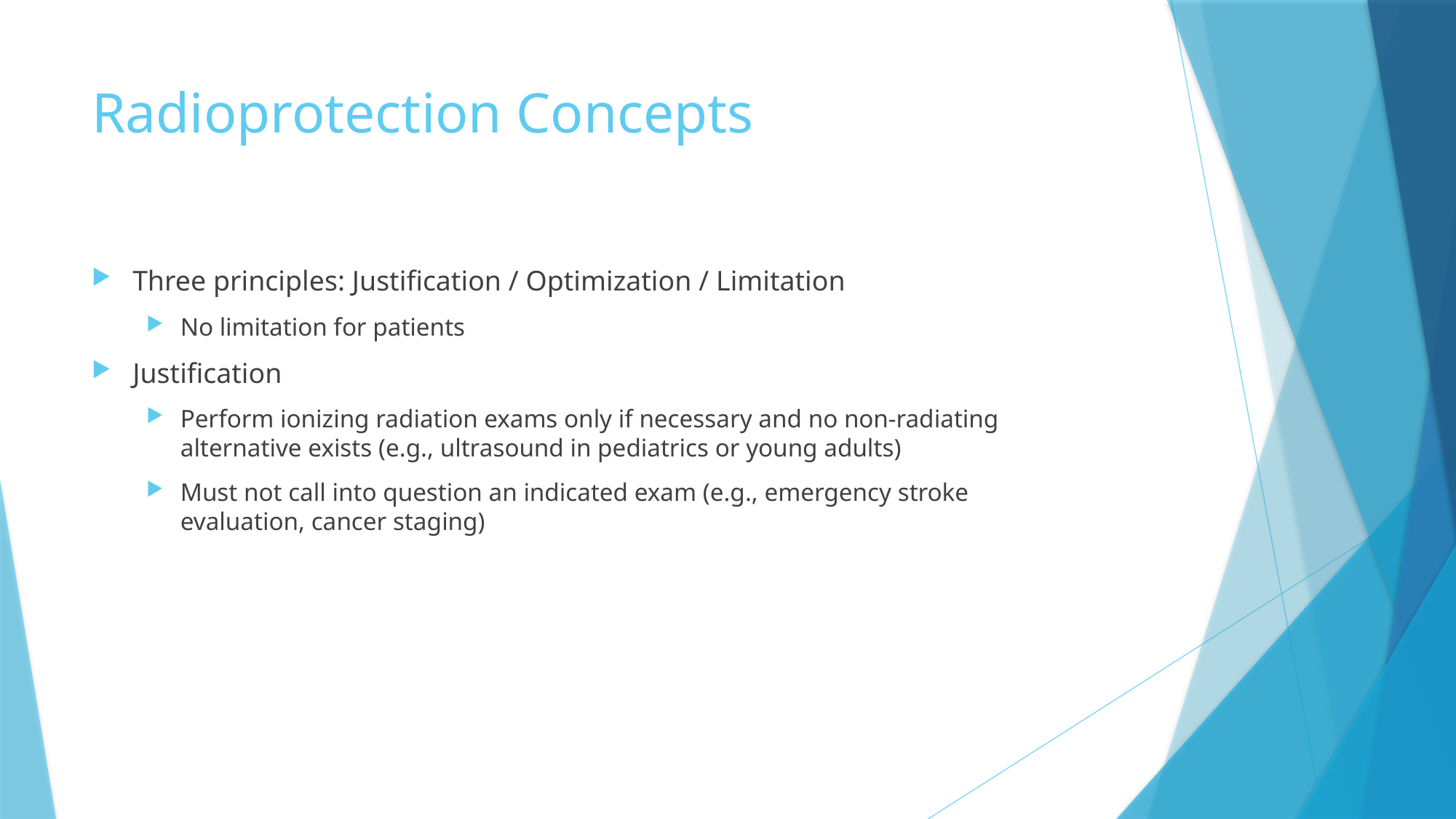

# Radioprotection Concepts
Three principles: Justification / Optimization / Limitation
No limitation for patients
Justification
Perform ionizing radiation exams only if necessary and no non-radiating alternative exists (e.g., ultrasound in pediatrics or young adults)
Must not call into question an indicated exam (e.g., emergency stroke evaluation, cancer staging)

## Slide 11
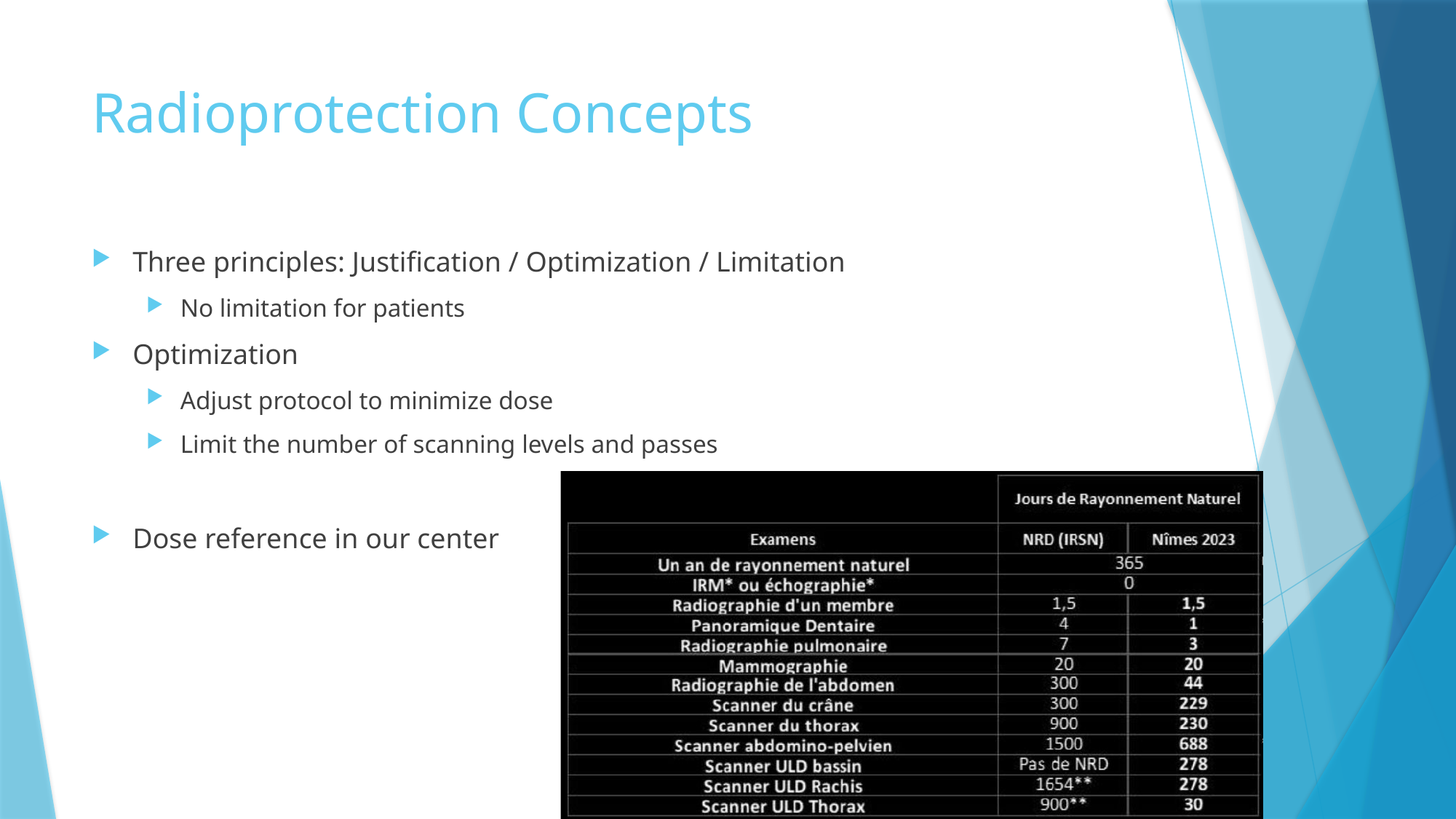

# Radioprotection Concepts
Three principles: Justification / Optimization / Limitation
No limitation for patients
Optimization
Adjust protocol to minimize dose
Limit the number of scanning levels and passes
Dose reference in our center

## Slide 12
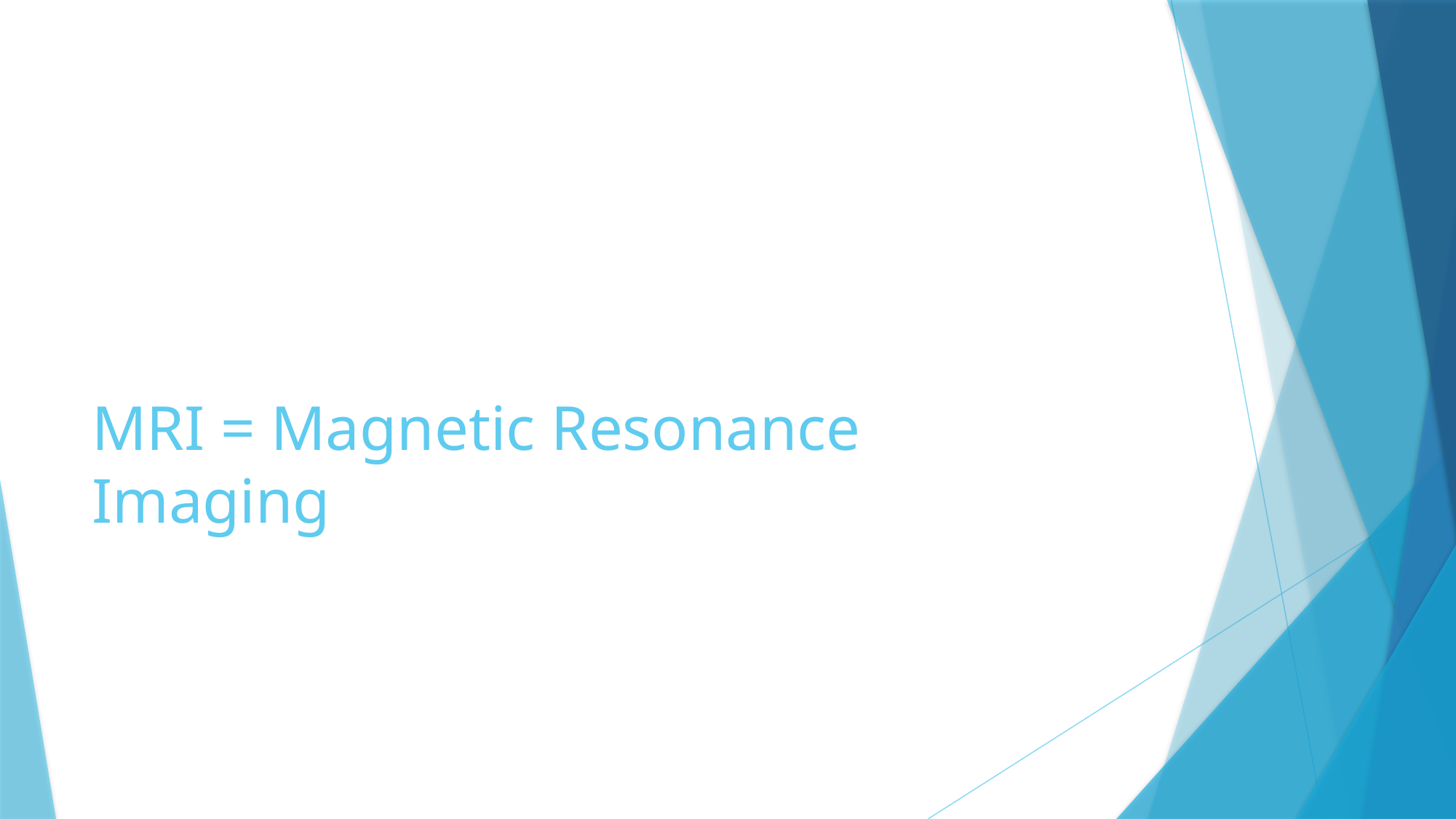

# MRI = Magnetic Resonance Imaging

## Slide 13
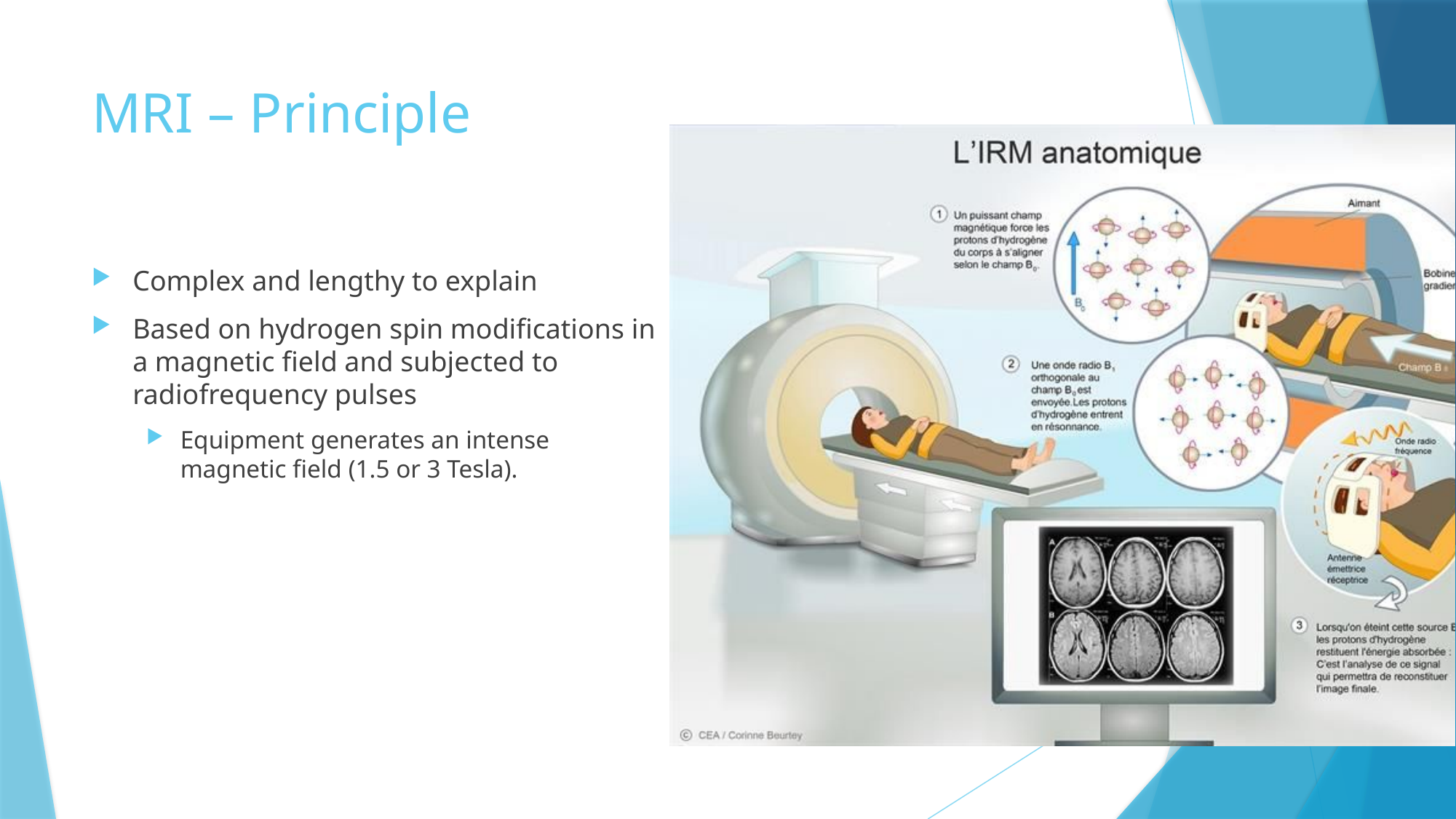

# MRI – Principle
Complex and lengthy to explain
Based on hydrogen spin modifications in a magnetic field and subjected to radiofrequency pulses
Equipment generates an intense magnetic field (1.5 or 3 Tesla).

## Slide 14
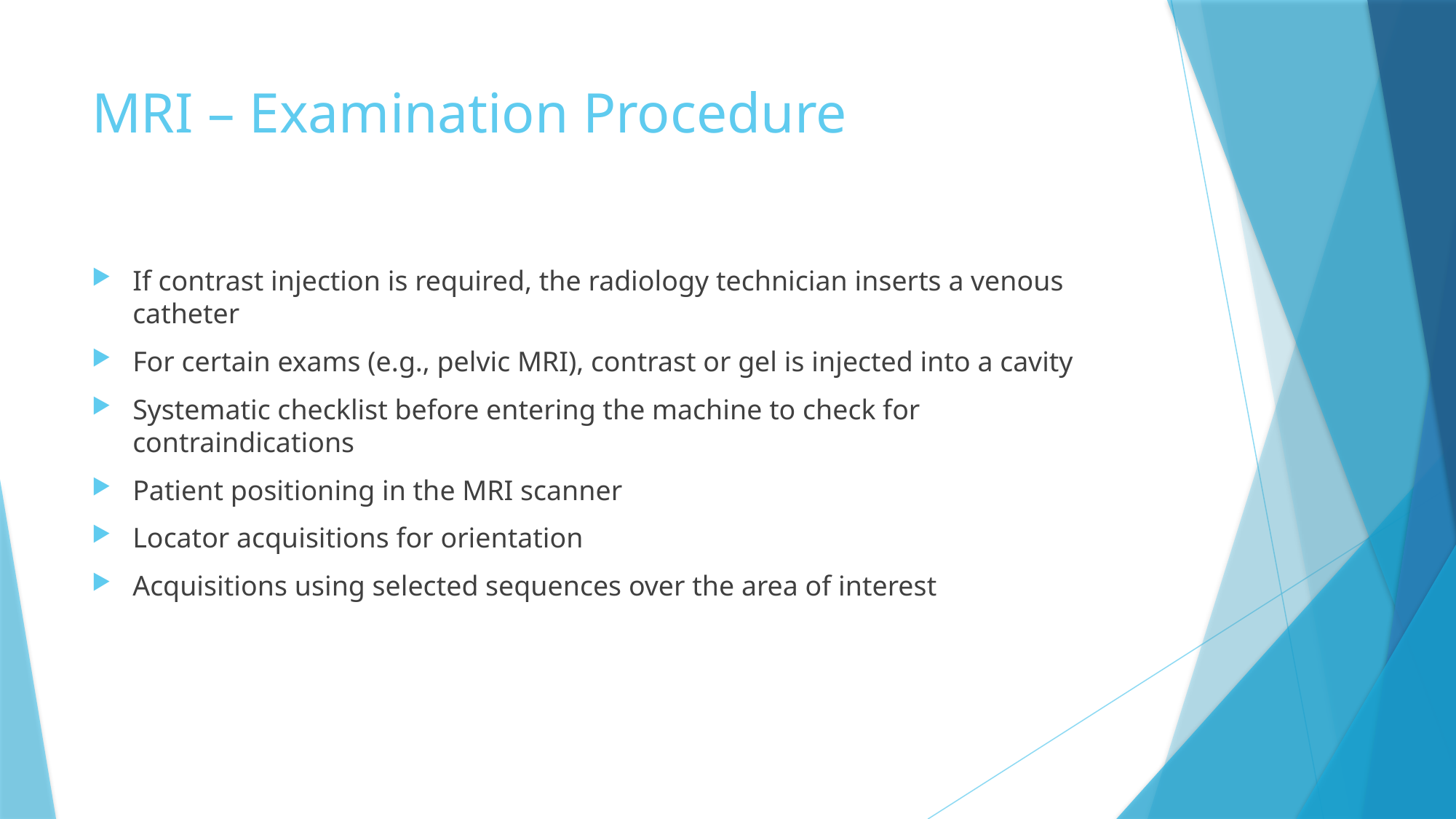

# MRI – Examination Procedure
If contrast injection is required, the radiology technician inserts a venous catheter
For certain exams (e.g., pelvic MRI), contrast or gel is injected into a cavity
Systematic checklist before entering the machine to check for contraindications
Patient positioning in the MRI scanner
Locator acquisitions for orientation
Acquisitions using selected sequences over the area of interest

## Slide 15
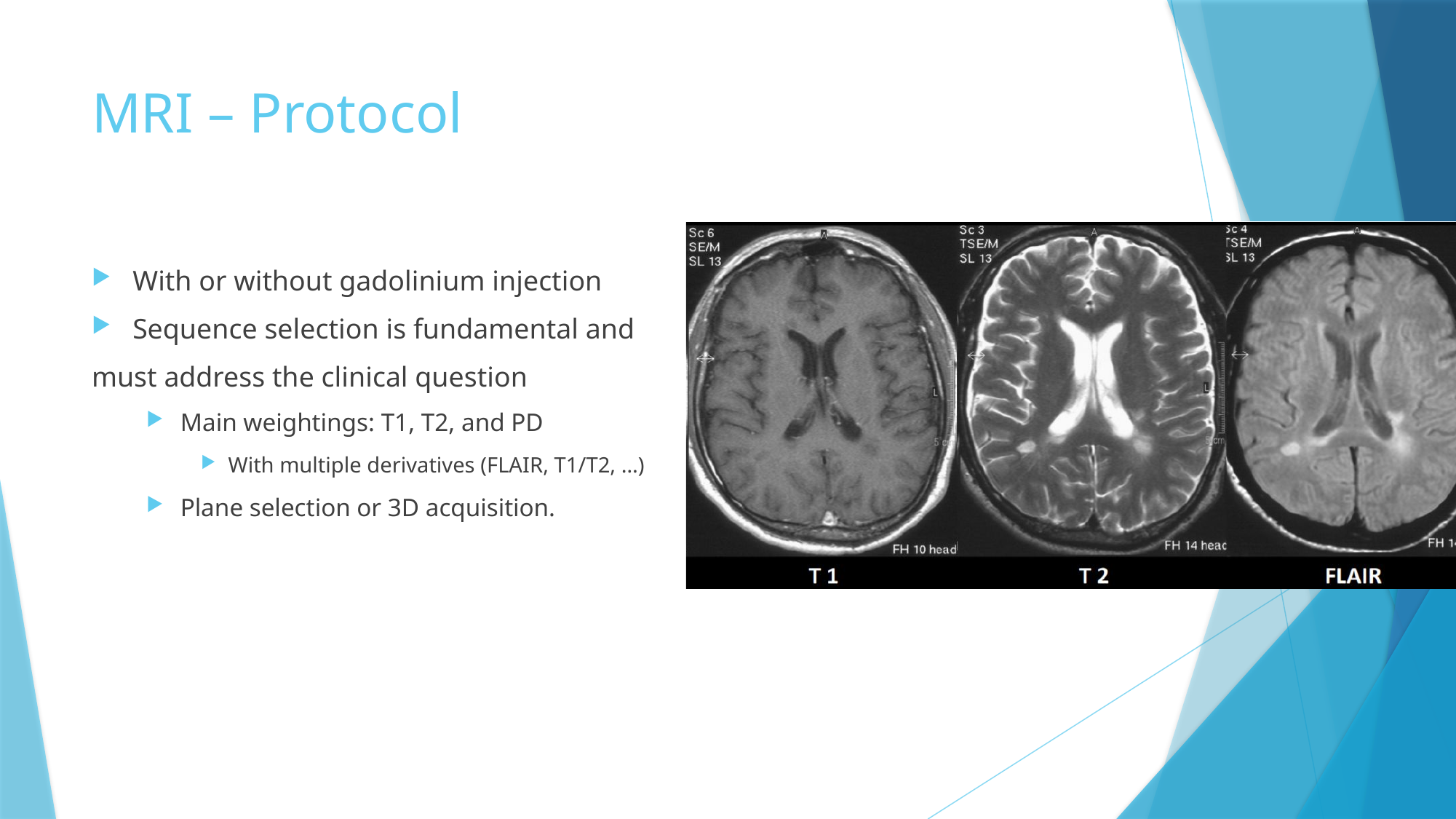

# MRI – Protocol
With or without gadolinium injection
Sequence selection is fundamental and
must address the clinical question
Main weightings: T1, T2, and PD
With multiple derivatives (FLAIR, T1/T2, …)
Plane selection or 3D acquisition.

## Slide 16
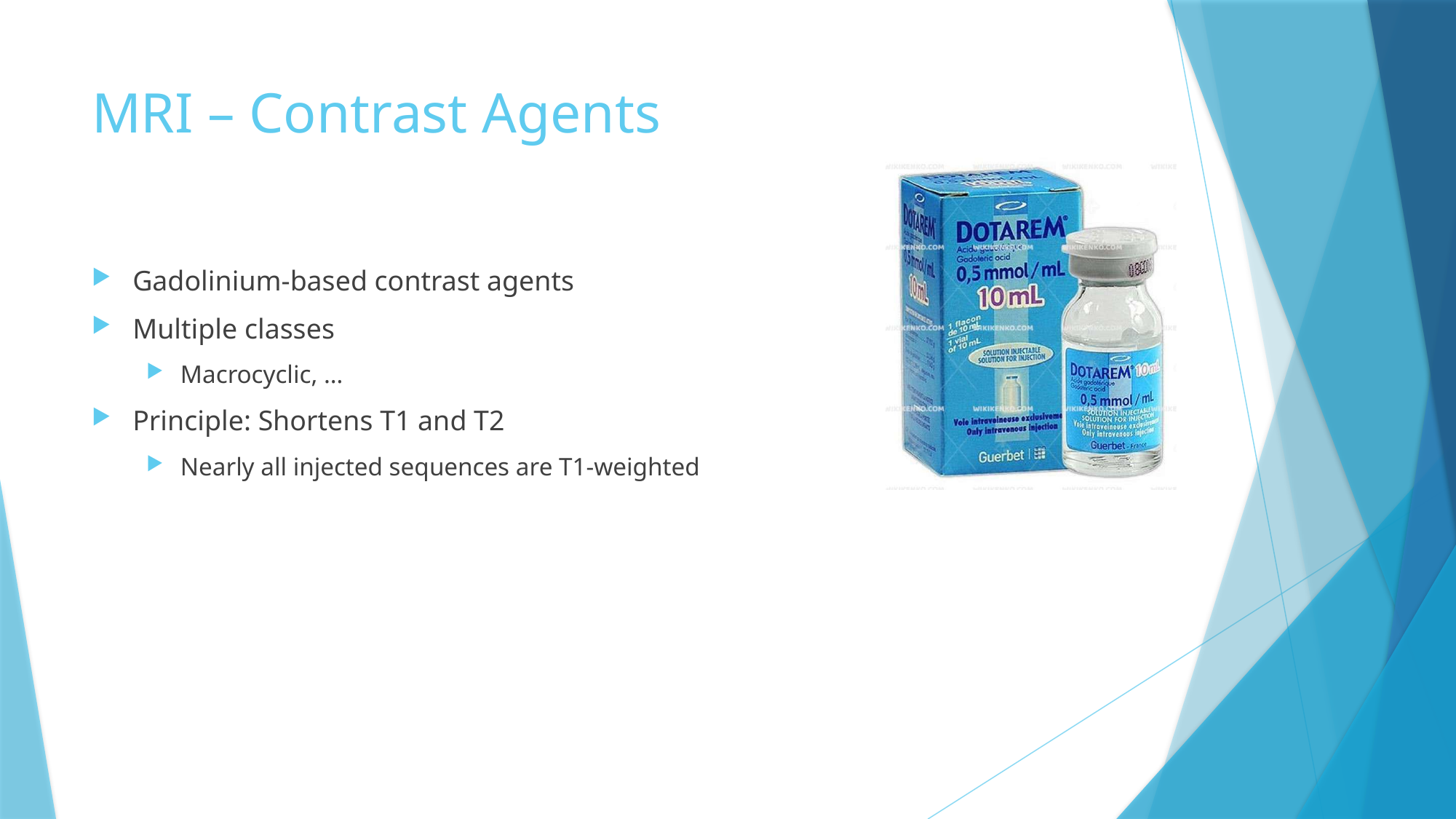

# MRI – Contrast Agents
Gadolinium-based contrast agents
Multiple classes
Macrocyclic, …
Principle: Shortens T1 and T2
Nearly all injected sequences are T1-weighted

## Slide 17
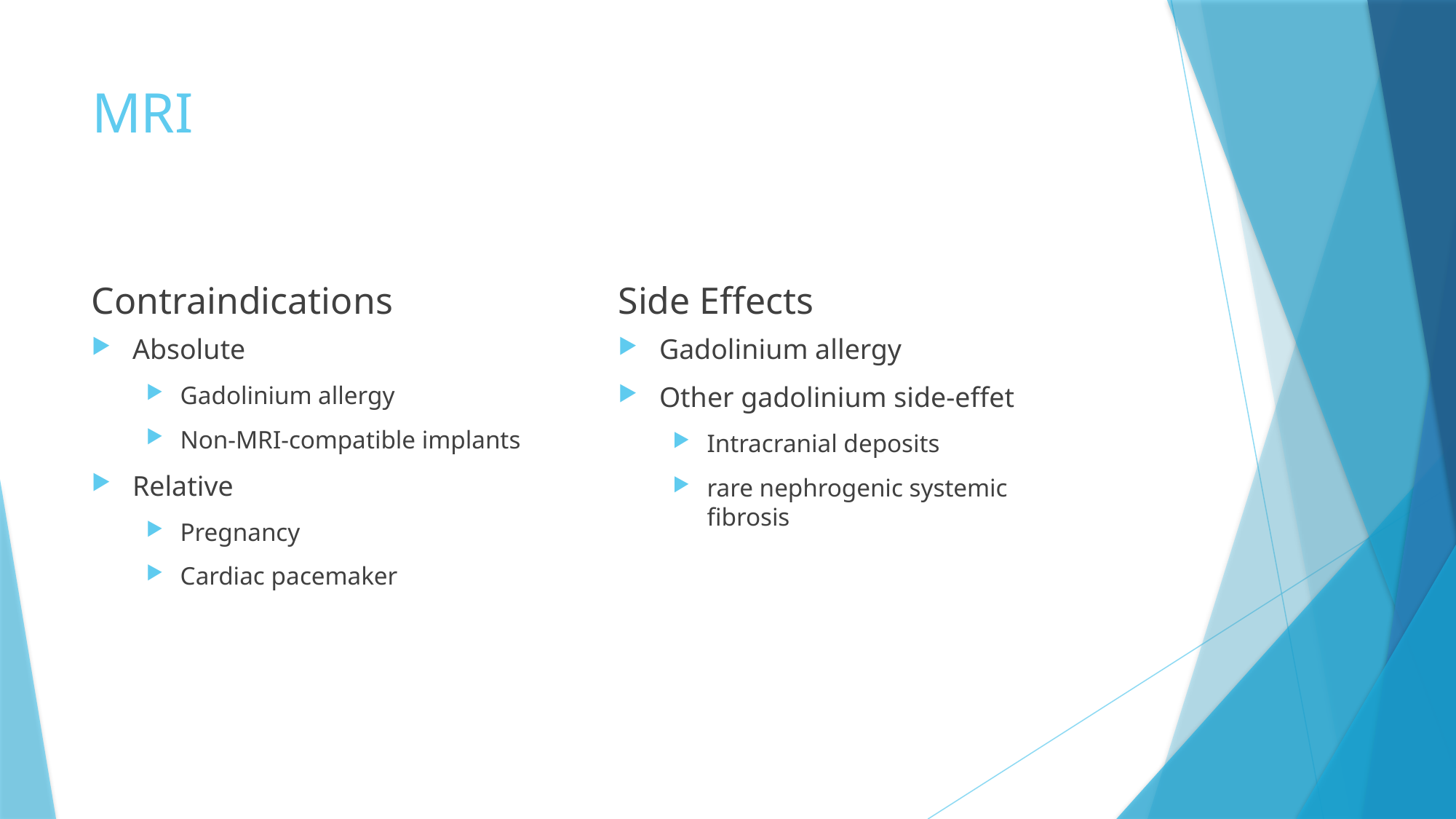

# MRI
Contraindications
Side Effects
Absolute
Gadolinium allergy
Non-MRI-compatible implants
Relative
Pregnancy
Cardiac pacemaker
Gadolinium allergy
Other gadolinium side-effet
Intracranial deposits
rare nephrogenic systemic fibrosis

## Slide 18
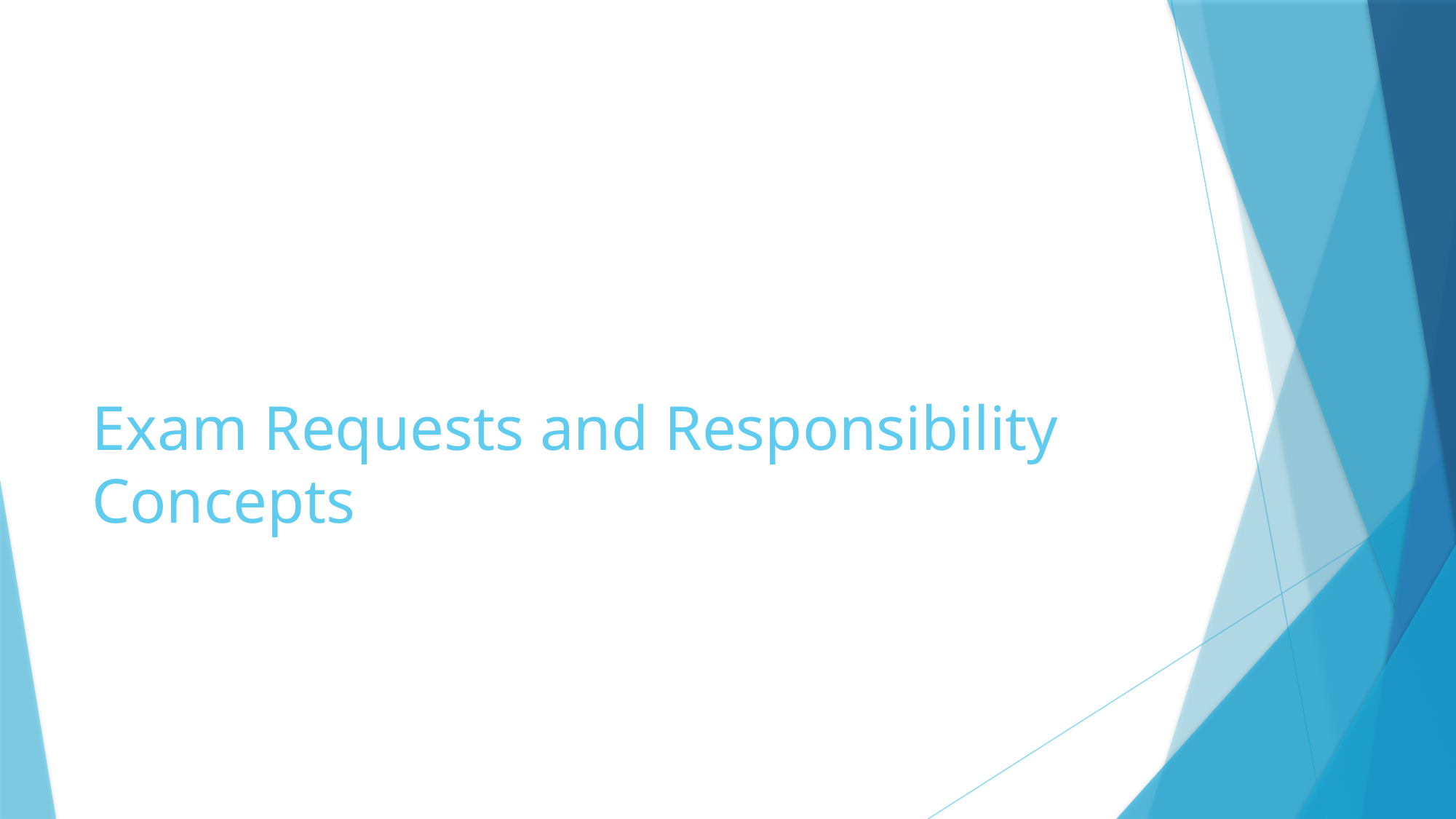

# Exam Requests and Responsibility Concepts

## Slide 19
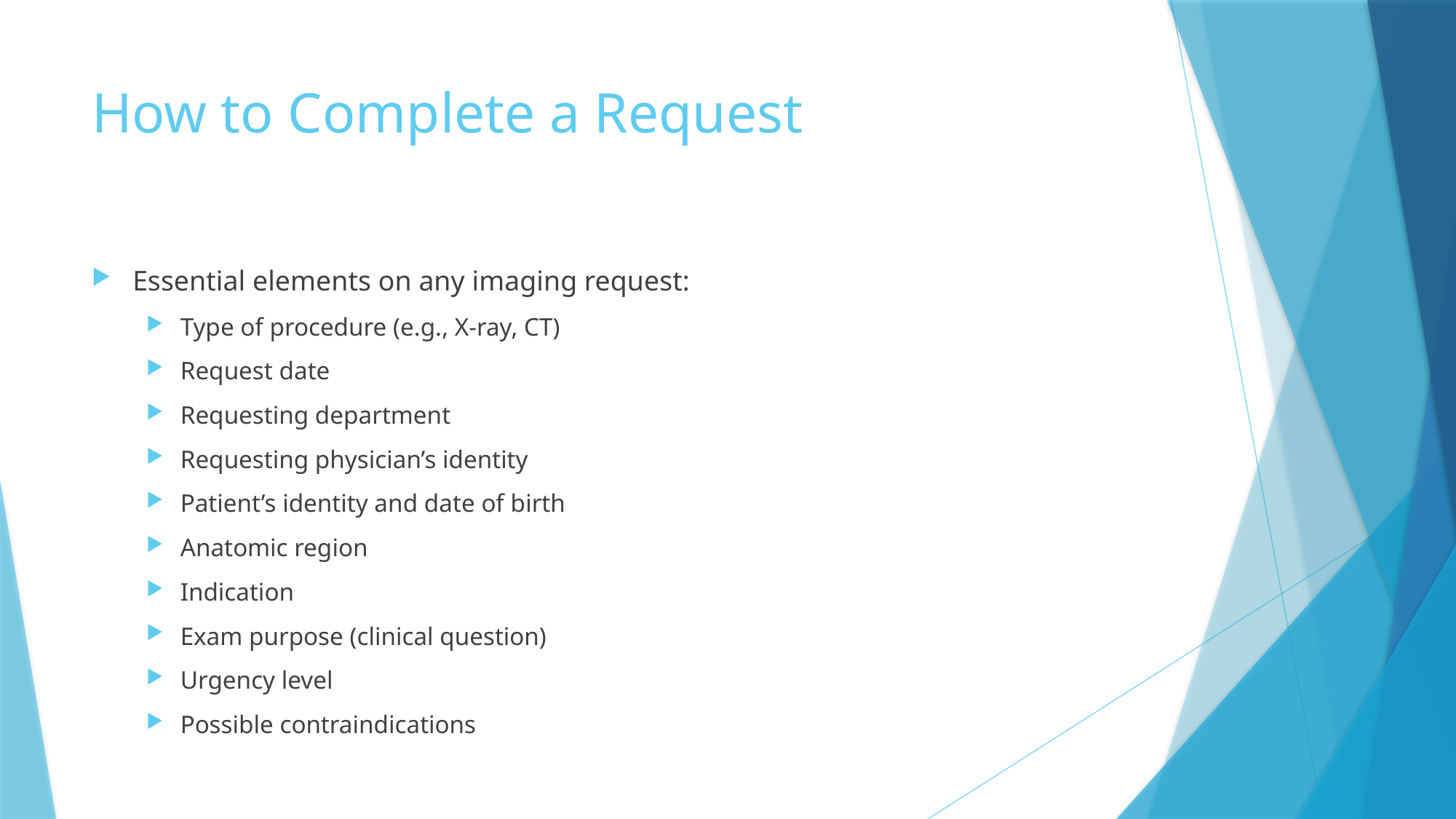

# How to Complete a Request
Essential elements on any imaging request:
Type of procedure (e.g., X-ray, CT)
Request date
Requesting department
Requesting physician’s identity
Patient’s identity and date of birth
Anatomic region
Indication
Exam purpose (clinical question)
Urgency level
Possible contraindications

## Slide 20
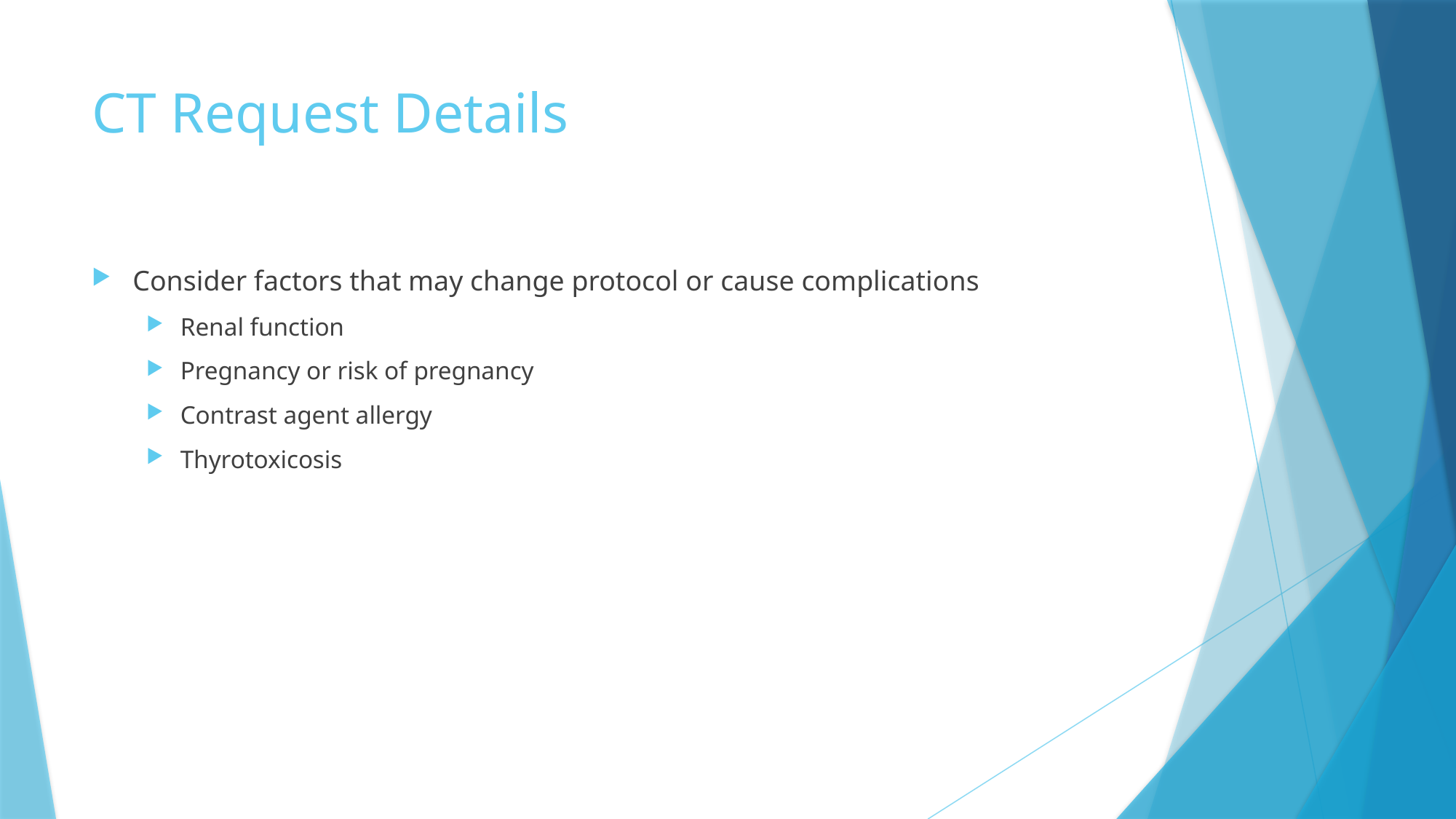

# CT Request Details
Consider factors that may change protocol or cause complications
Renal function
Pregnancy or risk of pregnancy
Contrast agent allergy
Thyrotoxicosis

## Slide 21
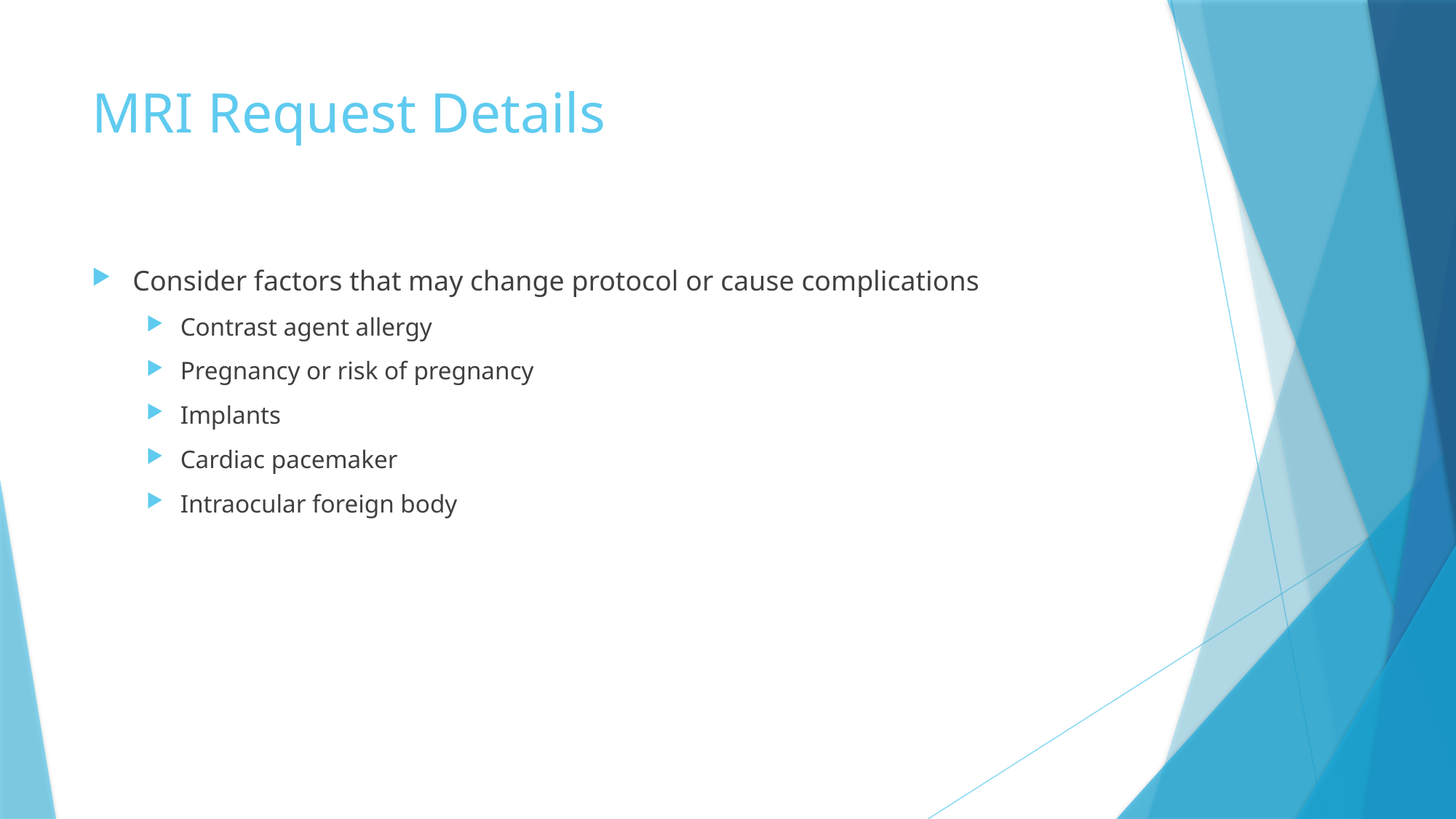

# MRI Request Details
Consider factors that may change protocol or cause complications
Contrast agent allergy
Pregnancy or risk of pregnancy
Implants
Cardiac pacemaker
Intraocular foreign body

## Slide 22
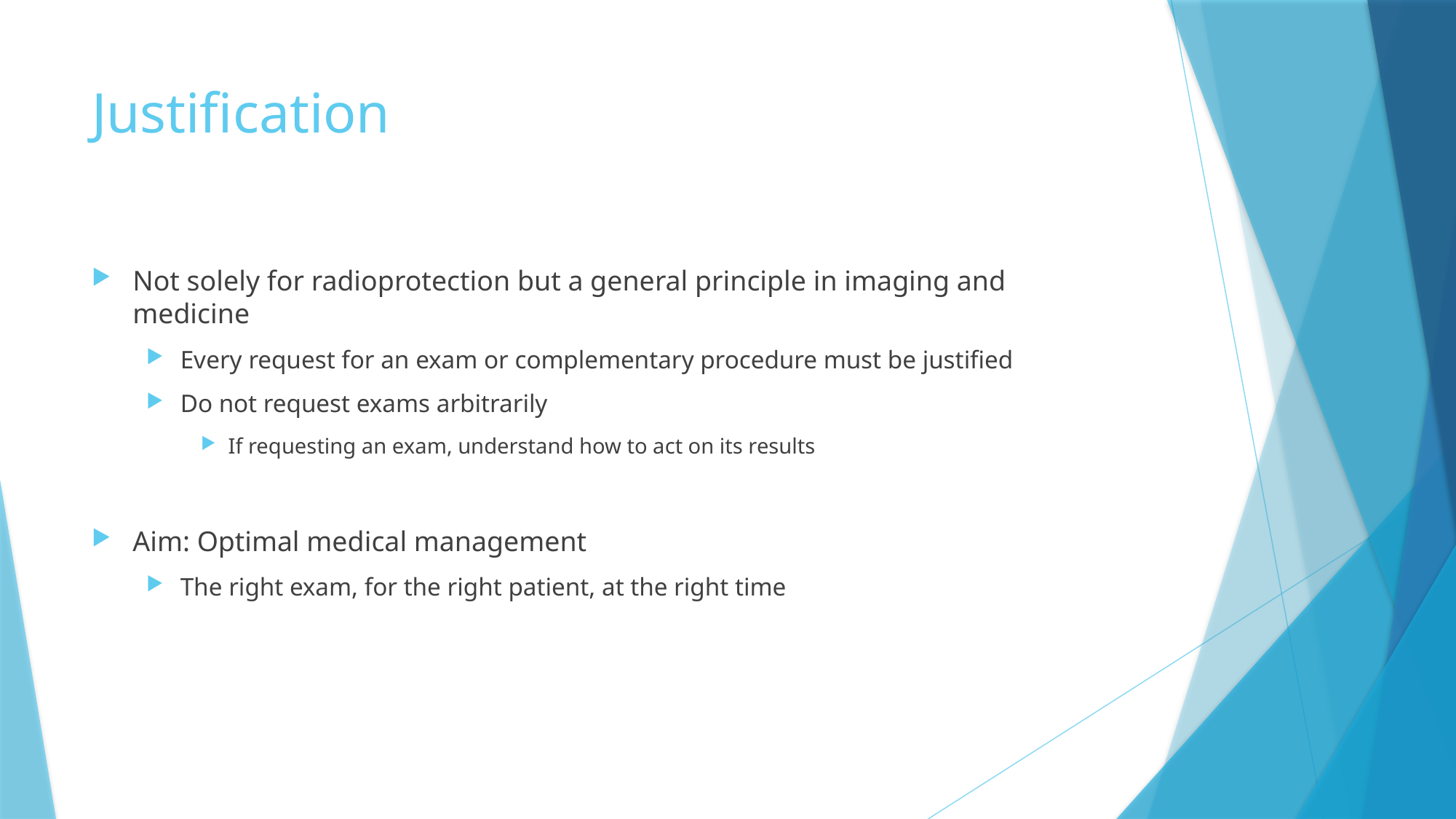

# Justification
Not solely for radioprotection but a general principle in imaging and medicine
Every request for an exam or complementary procedure must be justified
Do not request exams arbitrarily
If requesting an exam, understand how to act on its results
Aim: Optimal medical management
The right exam, for the right patient, at the right time

## Slide 23
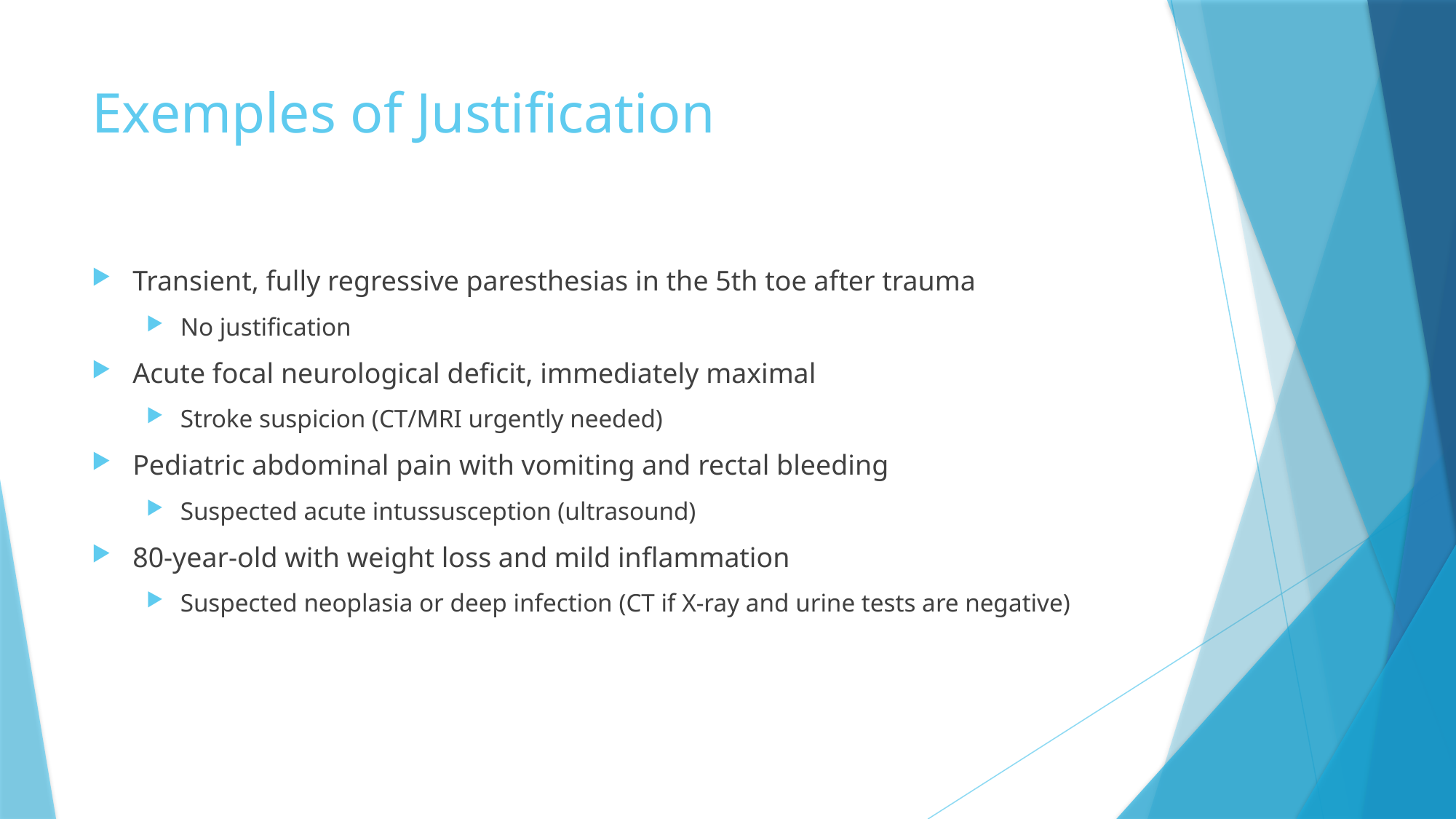

# Exemples of Justification
Transient, fully regressive paresthesias in the 5th toe after trauma
No justification
Acute focal neurological deficit, immediately maximal
Stroke suspicion (CT/MRI urgently needed)
Pediatric abdominal pain with vomiting and rectal bleeding
Suspected acute intussusception (ultrasound)
80-year-old with weight loss and mild inflammation
Suspected neoplasia or deep infection (CT if X-ray and urine tests are negative)

## Slide 24
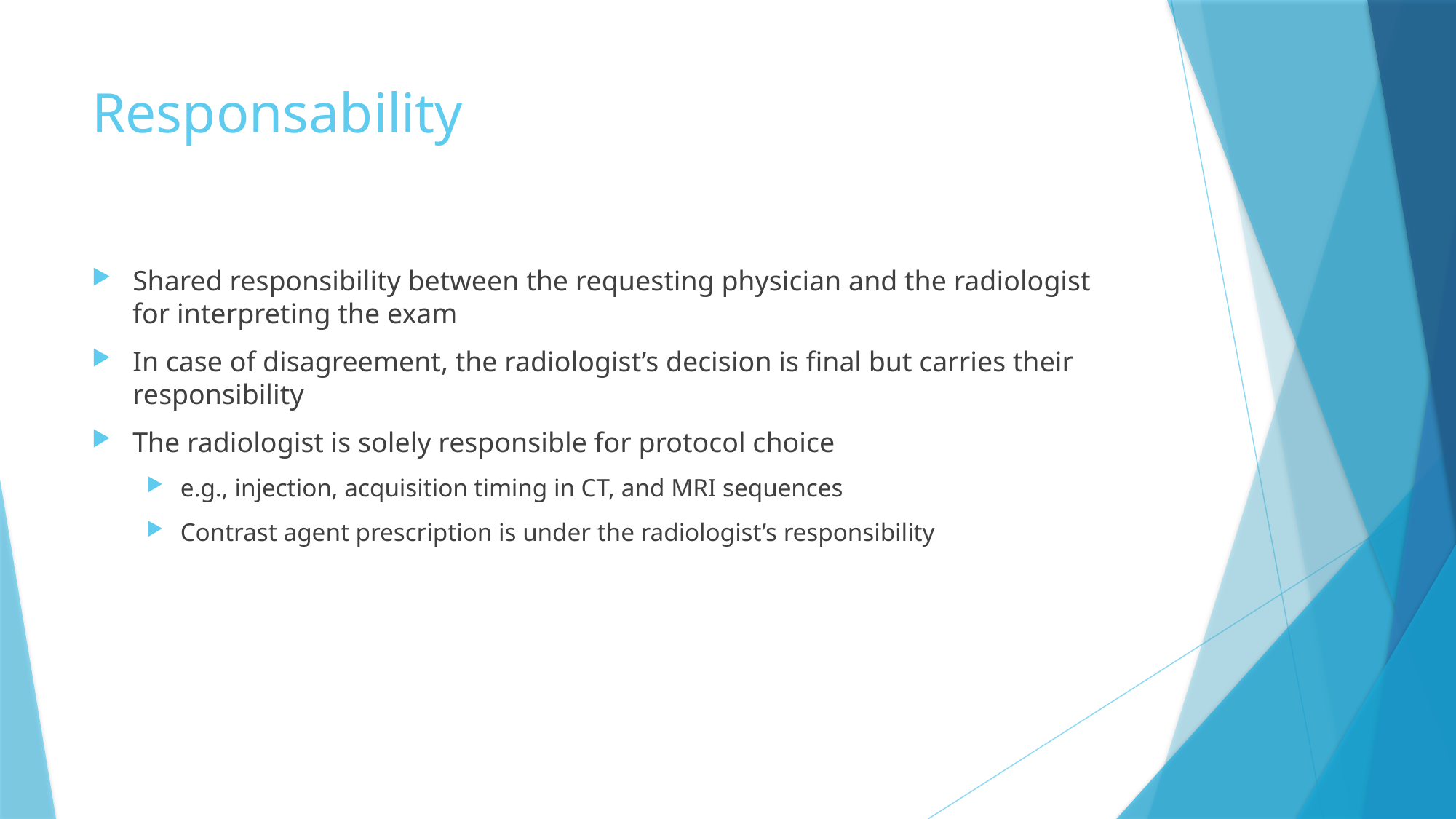

# Responsability
Shared responsibility between the requesting physician and the radiologist for interpreting the exam
In case of disagreement, the radiologist’s decision is final but carries their responsibility
The radiologist is solely responsible for protocol choice
e.g., injection, acquisition timing in CT, and MRI sequences
Contrast agent prescription is under the radiologist’s responsibility
