# Supplementary material for: Comparing active teaching to hybrid lecture-based method for learning radiology basics: A single center controlled study
Source: Res Diagn Interv Imaging. 2025 Mar 8;13:100054. doi: 10.1016/j.redii.2025.100054 (PMC11930576; doi:10.1016/j.redii.2025.100054)
Supplement: Supplementary file 2 [file mmc2.docx]

**Supplementary appendices**

Supplementary appendix 1: Student test

**Part 1 : Questions about the student and their background (only for the pre-test)**

- What is your gender?
- How old are you?
- How did you gain admission to the second year of medicine?
  - PASS
  - LAS
  - Other
- Have you obtained a degree between high school diploma and today?
  - Yes
  - No
- Do you already have an idea of the medical specialty you would like to pursue?
  - Yes
    - Which one ?
  - No
- Are you interested in the specialty of Radiodiagnosis and Medical Imaging?
  - Yes
  - No
- Have you already visited a medical imaging department?
  - Yes
  - No
- Do you have family working in this field?
  - Yes
  - No

**Part 2: Questions about medical imaging exams**

- How many patients have you seen during this session?
- Do you know how a CT scan (Computed Tomography) is performed?
  - Yes
  - No
- Do you know how an MRI (Magnetic Resonance Imaging) is performed?
  - Yes
  - No
- Name 5 elements that must be included in any request for medical imaging exams (regardless of the modality).
- Name 3 elements that could be contraindications or protocol modifiers specifically required in a CT scan request.
- Name 5 elements that could be contraindications or protocol modifiers specifically required in an MRI request.
- Name 3 adverse effects of iodinated contrast agents.
- How many months of natural radiation exposure does a brain CT scan in Nîmes correspond to?
  - 1 month
  - 4 months
  - 7 months
  - 10 months
  - 13 months
- A 73-year-old patient presents with a sudden neurological deficit, suggesting a stroke. Which exam(s) and within what timeframe do you believe it is appropriate to request?
  - Urgent brain CT-scan
  - Non urgent brain CT-scan
  - Urgent brain MRI
  - Non urgent brain MRI
- An 86-year-old patient has been experiencing a decline in general condition for several weeks with a mild inflammatory syndrome (CRP at 37 mg/L) without clear clinical indication. Which exam do you believe is relevant to request?
  - Urgent whole-body MRI
  - Semi-urgent whole-body MRI
  - Urgent thoraco-abdomino-pelvic CT scan
  - Semi-urgent thoraco-abdomino-pelvic CT scan
- In case of disagreement between the requesting physician and the radiologist regarding the exam to be performed, who makes the final decision?
  - Physician requesting the exam
  - Radiologist
- A young patient with a normal weight presents with symptoms of appendicitis. To minimize exposure to ionizing radiation, which exam do you consider the most appropriate?
  - Abdomino-pelvic CT-scan
  - Abdomino-pelvic MRI
  - Abdomino-pelvic US
- In the event of an adverse reaction following an injection of a contrast agent, who is the prescribing physician of the active ingredient and liable for the consequences?
  - Physician requesting the exam
  - Radiologist
- An intern in pediatric emergency is in charge of a 6-month-old child with abdominal pain, suspecting acute intestinal intussusception. The intern requests a CT scan. What do you think about the justification of this action?
  - It is justified as it is a surgical emergency.
  - It is justified as the CT scan will enable positive and differential diagnosis.
  - It is not justified as it is not the appropriate exam for this diagnosis.
  - It is not justified as there is an alternative that does not use X-rays.
- A 24-year-old patient presents with febrile abdominal pain. The radiologist decides to perform a single portal phase acquisition instead of a multiphase acquisition. What radioprotection principle does this refer to?
  - Limitation
  - Optimization
  - Justification
- Are you satisfied with this training session?
  - Scale from 0 to 10
- Do you believe this session has been useful for your training?
  - Scale from 0 to 10

Supplementary appendix 2: Imaging Exam Appropriation Form

**Patient Identification**

- Is the patient's name / first name / date of birth present on the exam request?
  - Yes
  - No

**Exam Request Information**

- Is the name of the requesting physician present?
  - Yes
  - No
- Is the name of the requesting department or a contact number provided?
  - Yes
  - No
- Is the indication and purpose clearly specified?
  - Yes
  - No
- Is the exam request form filled out completely?
  - Yes
  - No

**Modality**

- What is the modality of the exam?
  - CT scan
  - MRI

**Indication and Relevance**

- Why is the medical imaging examination being performed?
- On a scale of 0 to 10, how do you rate the relevance of the examination?
  - Scale 0 to 10
- What was the requested timeframe for the examination?
  - Urgent
  - Semi-urgent
  - Non-urgent
- Do you find the requested timeframe to be appropriate?
  - Scale 0 to 10
- What is the context of the examination request?
  - Screening
  - Diagnosis
  - Oncological follow-up
  - Postoperative follow-up
  - Other
- What are the formal contraindications for this examination?
- What are the relative contraindications for this examination?

**Contrast Agent**

- Was a contrast agent injected?
  - Yes
  - No
- If contrast agent was injected, what product was used
- What are the relative contraindications for the injection of this contrast agent?
- What are the absolute contraindications for the injection of this contrast agent?
- What are the possible adverse effects of the contrast agent used?

**Dosimetry**

- Does the examination expose to ionizing radiation?
  - Yes
  - No
- If exposure, how many days of natural radiation does the dose correspond to?

**Results and Treatment**

- Did this examination lead to a modification of therapeutic care?
  - Yes
  - No
- How do you assess the relevance of the examination for the patient's therapeutic management?
  - Scale 0 to 10

**Patient and Experience**

- Was the patient informed about the procedure of the examination?
  - Yes
  - No
  - Partially
  - Did not want to know
  - Not relevant
- Did the patient understand the relevance of the examination for their treatment?
  - Scale 0 to 10
- What is the level of patient's anxiety on a scale of 0 to 10 before the examination?
  - Scale 0 to 10
- What is the level of patient's anxiety on a scale of 0 to 10 after the examination?
  - Scale 0 to 10
- Did the patient experience any pain during the examination?
  - Scale 0 to 10

Supplementary appendix 3: score correction

Correct answers are bold for MCQ and SCQ.

SCQ were corrected with 0 points for false answers or 1 point for correct answers.

MCQ were corrected by 1 point if all correct elements were selected and 0.5 points have been deducted for each incorrect selection or correct option not selected.

- Do you know how a CT scan (Computed Tomography) is performed? (1 point)
  - **Yes**
  - No
- Do you know how an MRI (Magnetic Resonance Imaging) is performed? (1 point)
  - **Yes**
  - No
- Name 5 elements that must be included in any request for medical imaging exams (regardless of the modality). (0.7 points for each correct answer, up to a maximum of 3.5 points)
  - Five elements among
    - Type of procedure (X-ray, CT scan, etc.)
    - Date of the request
    - Requesting yard
    - Name of the requesting physician
    - Patient's identity
    - Patient's date of birth
    - Anatomical region
    - Medical indication
    - Purpose of the examination (question asked)
    - Degree of emergency
    - Contraindications
- Name 3 elements that could be contraindications or protocol modifiers specifically required in a CT scan request. (0.5 points for each correct answer, up to a maximum of 1.5 points)
  - 3 elements among
    - Renal function
    - Pregnancy or potential pregnancy risk, if applicable
    - Allergy to contrast agent
    - Thyrotoxicosis
- Name 5 elements that could be contraindications or protocol modifiers specifically required in an MRI request. (0.5 points for each correct answer, up to a maximum of 2.5 points)
  - 5 elements among
    - Allergy to contrast agent
    - Pregnancy or potential pregnancy risk
    - Implantable material
    - Cardiac stimulation material
    - Intraocular foreign body
    - Claustrophobia
- Name 3 adverse effects of iodinated contrast agents. (0.5 points for each correct answer, up to a maximum of 1.5 points)
  - 3 elements among
    - Allergy
    - Acute renal failure
    - Thyroid dysfunction
    - Acute pulmonary oedema
- How many months of natural radiation exposure does a brain CT scan in Nîmes correspond to? (1 point)
  - 1 month
  - 4 months
  - **7 months**
  - 10 months
  - 13 months
- A 73-year-old patient presents with a sudden neurological deficit, suggesting a stroke. Which exam(s) and within what timeframe do you believe it is appropriate to request? (1 point)
  - **Urgent brain CT-scan**
  - Non urgent brain CT-scan
  - **Urgent brain MRI**
  - Non urgent brain MRI
- An 86-year-old patient has been experiencing a decline in general condition for several weeks with a mild inflammatory syndrome (CRP at 37 mg/L) without clear clinical indication. Which exam do you believe is relevant to request? (1 point)
  - Urgent whole-body MRI
  - Semi-urgent whole-body MRI
  - Urgent thoraco-abdomino-pelvic CT scan
  - **Semi-urgent thoraco-abdomino-pelvic CT scan**
- In case of disagreement between the requesting physician and the radiologist regarding the exam to be performed, who makes the final decision? (2 points)
  - Physician requesting the exam
  - **Radiologist**
- A young patient with a normal weight presents with symptoms of appendicitis. To minimize exposure to ionizing radiation, which exam do you consider the most appropriate? (1 point)
  - Abdomino-pelvic CT-scan
  - Abdomino-pelvic MRI
  - **Abdomino-pelvic US**
- In the event of an adverse reaction following an injection of a contrast agent, who is the prescribing physician of the active ingredient and liable for the consequences? (1 point)
  - Physician requesting the exam
  - **Radiologist**
- An intern in pediatric emergency is in charge of a 6-month-old child with abdominal pain, suspecting acute intestinal intussusception. The intern requests a CT scan. What do you think about the justification of this action? (1 point)
  - It is justified as it is a surgical emergency.
  - It is justified as the CT scan will enable positive and differential diagnosis.
  - **It is not justified as it is not the appropriate exam for this diagnosis.**
  - **It is not justified as there is an alternative that does not use X-rays.**
- A young 24-year-old patient presents with febrile abdominal pain. The radiologist decides to perform a single portal phase acquisition instead of a multiphase acquisition. What radioprotection principle does this refer to? (1 point)
  - Limitation
  - **Optimization**
  - Justification
